# Temperature variation generates interspecific synchrony but spatial asynchrony in survival for freshwater fish communities

**DOI:** 10.1002/ece3.10700

**Published:** 2023-11-12

**Authors:** Kasey C. Pregler, Xinyi Lu, George P. Valentine, Seoghyun Kim, Yoichiro Kanno

**Affiliations:** ^1^ Department of Fish, Wildlife, and Conservation Biology Colorado State University Fort Collins Colorado USA; ^2^ Department of Forestry and Environmental Conservation Clemson University Clemson South Carolina USA; ^3^ Graduate Degree Program in Ecology Colorado State University Fort Collins Colorado USA

**Keywords:** asynchrony, demographic variation, environmental drivers, freshwater fish, survival, synchrony

## Abstract

Identifying environmental drivers of demographic variation is key to predicting community‐level impacts in response to global change. Climate conditions can synchronize population trends and can occur both spatially for populations of the same species, and across multiple species within the same local community. The aim of this study was to investigate patterns of temporal variation in survival for freshwater fish communities in two geographically close but isolated sites and to understand the amount of variation accounted for by abiotic covariates including metrics of water temperature and stream flow. Using mark‐recapture data, we estimated bi‐monthly apparent survival in a Bayesian Cormack‐Jolly‐Seber framework. The model included random effects to quantify temporal variance to understand species synchrony with the rest of the fish community and between sites. Study species included bluehead chub (*Nocomis leptocephalus*), creek chub (*Semotilus atromaculatus*), and striped jumprock (*Moxostoma rupiscartes*) in the southeastern USA. Results showed that survival varied over time and periods of low survival were associated with higher mean water temperature. However, temporal patterns of survival differed among species and between sites, where survival was synchronous among species within a site but asynchronous between sites for the same species despite their spatial proximity. Study streams differed in summer thermal regimes, which resulted in contrasting summer survival patterns, suggesting sensitivity of these fishes to warming. We found that interspecific synchrony was greater than spatial synchrony, where regional drivers such as temperature may interact with local habitat leading to differences in survival patterns at fine spatial scales. Finally, these findings show that changes in the timing and magnitude of environmental conditions can be critical in limiting vital rates and that some populations may be more resilient to climate variation than others.

## Graphical abstract

Climate conditions can synchronize population trends and can occur both spatially for populations of the same species, and across multiple species within the same local community. Here, we quantified the effects of environmental drivers on both interspecific and spatial synchrony in survival for two stream fish communities in geographically close but isolated sites using mark‐recapture methods. We found that high summer temperatures synchronized species' survival within a community but were spatially asynchronous between sites, where the degree of interspecific synchrony differed between the two streams due to interactions between local habitat differences and summer temperature regimes.

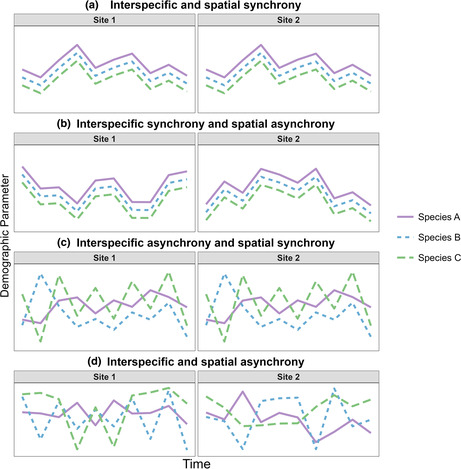

## 1 INTRODUCTION

In a time of global change, identifying patterns of demographic variation is a key aim in elucidating mechanisms of demographic change and predicting population‐ and community‐level impacts (Muths et al., 2017). Temporal fluctuations in populations and their environments are widespread in natural systems (Loreau & de Mazancourt, 2008), and researchers have investigated the mechanisms driving demographic variation at different scales including among populations within a species and among species within a community (Cayuela et al., 2016; Koenig, 2001; Trenham et al., 2003). The degree of correlation in temporal trends, such as abundance, survival, or other demographic metrics, among species or populations indicates the amount of synchrony (i.e., high correlation, either positive or negative) or asynchrony (i.e., low correlation; Liebhold et al., 2004). Synchrony and asynchrony have been studied in a variety of contexts that are relevant in understanding population responses to environmental change, community structure, and conservation applications such as extinction risk (Bino et al., 2020; Vendrametto Granzotti et al., 2021; Walther, 2010).

The degree of synchrony and asynchrony in a system can have important consequences for both extinction dynamics and ecosystem stability (Walter et al., 2021). High amounts of synchrony among populations can make species vulnerable to extinction when experiencing shared stressors (e.g., climate change, Palmqvist & Lundberg, 1998). However, high amounts of synchrony among populations are not necessarily always associated with extinction. For example, extinction risk can be low among synchronized populations if they are connected, exhibit high dispersal and population growth rates, and have large habitat size (Matter, 2001). But spatial asynchrony among populations can act as a stabilizing mechanism thereby decreasing extinction risk and buffer both populations and communities against environmental change (i.e., the insurance hypothesis, portfolio effect; Cline et al., 2022; Hammond et al., 2020; Schindler et al., 2015). For example, asynchrony among connected populations (i.e., metapopulations) allows dispersal of individuals from robust populations to demographically rescue or recolonize declining and extirpated populations (Brown & Kodric‐Brown, 1977). For sympatric species in a community, high amounts of interspecific synchrony can decrease community stability and in turn lead to increased extinction risk (Elmqvist et al., 2003; Mori et al., 2013). Asynchrony among species within a community can facilitate species' coexistence by relaxing interspecific competition (Siepielski & McPeek, 2010) and result in community stability through species tradeoffs such that a decline in one species is compensated by a rise in another (Loreau & de Mazancourt, 2008; Walter et al., 2021).

The amount of synchrony is regulated by a number of mechanisms including climate, trophic structure (e.g., predator–prey dynamics), dispersal, and biocomplexity (Kendall et al., 2000; Koenig, 1999; Robertson et al., 2015; Tavecchia et al., 2008). Climate and seasonal weather patterns can drive regional spatial synchrony across multiple populations for a given species (i.e., Moran effect, Moran, 1953). Individuals of neighboring populations are more likely to experience the same climatic drivers, with synchrony decreasing with distance (Kendall et al., 2000; Paradis et al., 1999). Increased synchrony among connected populations has also been linked to high amounts of dispersal that results in gene flow or density‐dependent processes (Ranta et al., 1995). Synchrony can also be influenced by similar life history traits that correlate with the environment such as reproduction timing and temperature (Chevalier et al., 2014; Tedesco & Hugueny, 2006) or tracking and competition for food resources or space (Hinks et al., 2015; Huitu et al., 2004). Conversely, asynchronous dynamics can arise with increasing amounts of biocomplexity in a system such as phenotypic diversity (e.g., life history diversity) and local adaptations in response to spatial heterogeneity in habitat (Hilborn et al., 2003; Rogers & Schindler, 2008). Additionally, neighboring populations that are isolated may have a higher probability of asynchrony if they do not respond similarly to climatic drivers and there is a lack of dispersal (Adler, 1994; Dibner et al., 2019).

**FIGURE 1:**
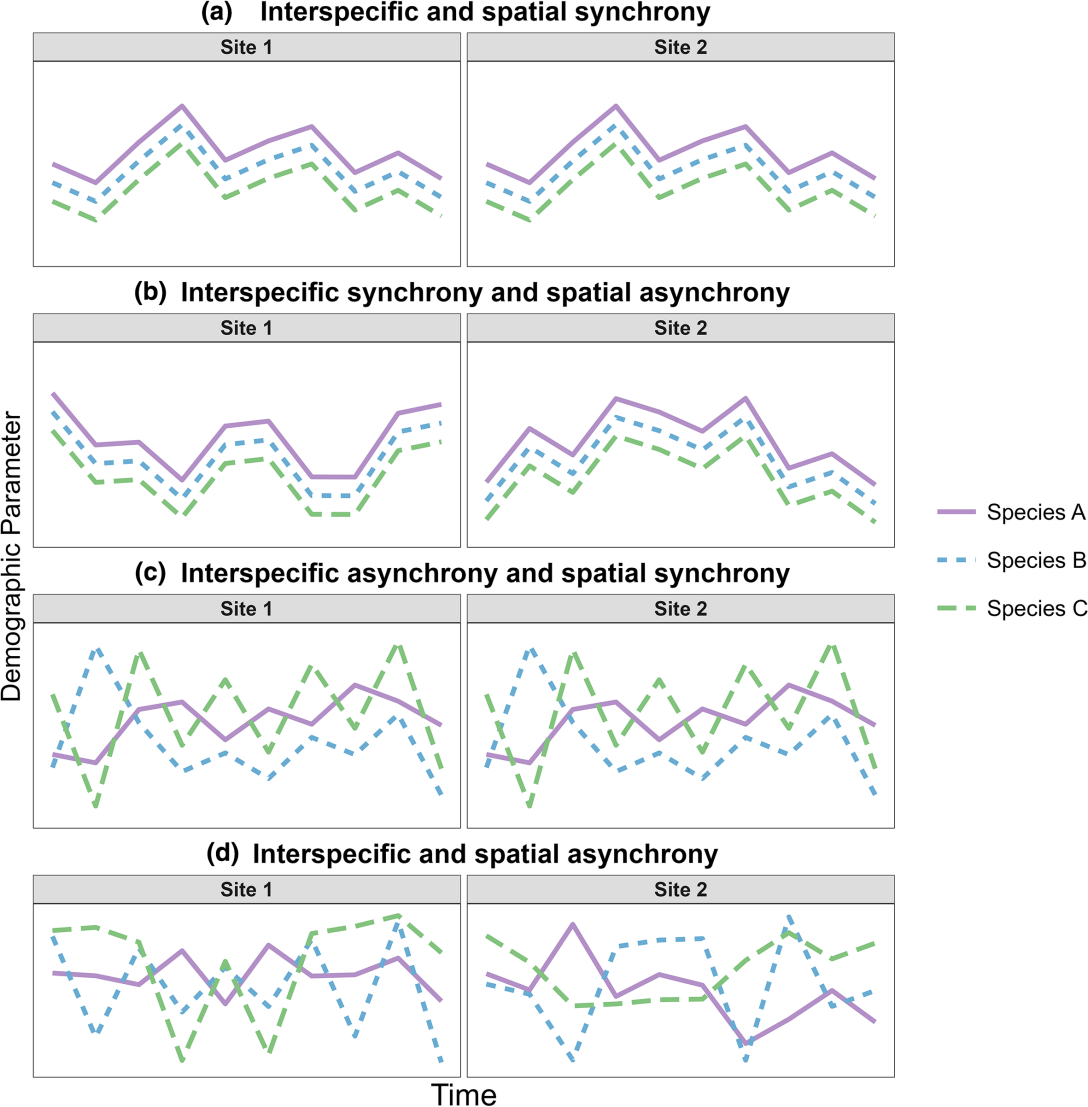
Conceptual diagram depicting four potential patterns of interspecific and spatial synchrony/asynchrony for a given demographic parameter. (a) Scenario 1. Interspecific and spatial synchrony where a demographic parameter is synchronous among species within a community and also for species' populations between sites. (b) Scenario 2. Interspecific synchrony and spatial asynchrony where a demographic parameter is synchronous among species within a community but asynchronous for species' populations between sites. (c) Scenario 3. Interspecific asynchrony and spatial synchrony where a demographic parameter is asynchronous over time among species within a community but synchronous for species' populations between sites. (d) Scenario 4. Interspecific and spatial asynchrony where a demographic parameter is asynchronous among species within a community and also for species' populations between sites.

Investigations into patterns of demographic variation have generally focused on spatial synchrony among geographically distinct populations of the same species (Bouchard et al., 2022; Grosbois et al., 2009; Olmos et al., 2020), but little attention has been paid to synchrony among different species within a community (i.e., interspecific synchrony; Raimondo et al., 2004; but see Lahoz‐Monfort et al., 2011, 2013, and Swallow et al., 2016). Species within a local community (i.e., interspecific synchrony) are exposed to similar biotic and abiotic environmental conditions and synchronization among different species within the same habitat can be influenced by shared stochastic effects such as weather and climate (Hansen et al., 2013), or shared predators (Raimondo et al., 2004; Vasseur & Fox, 2007). However, sympatric species may show asynchronous abundance patterns over time leading to high community turnover rates (Ives et al., 2003; Shimadzu et al., 2015), especially when community members are ecologically diverse and respond heterogeneously to environmental drivers (Haddad et al., 2008; Hordley et al., 2021). Similarly, spatial heterogeneity in the landscape influences the degree of demographic synchrony among sites, where the impacts of regional drivers such as climate may interact with local habitat characteristics to generate spatial asynchrony in demography (i.e., cross‐scale interactions) (Heffernan et al., 2014). Taken together, we theorize that patterns of interspecific and spatial synchrony are categorized into four scenarios (Figure 1), depending on whether synchrony or asynchrony occurs both interspecifically and spatially, or it occurs either interspecifically or spatially. The relative degree of interspecific and spatial synchrony has been little studied despite its importance in projecting community patterns and dynamics over space.

To understand patterns of interspecific and spatial synchrony in population dynamics we examined stream fish communities in the southeastern United States. While dispersal and biotic interactions can be important drivers of synchrony, the abiotic environment plays a key role in influencing fish demography (Beisner et al., 2006; Bond et al., 2015; Jackson et al., 2001). As ectotherms, fish physiology is closely tied to their abiotic environment, and variables such as water temperature can limit species' distributions and vital rates (Little et al., 2020). For fishes that inhabit lotic environments (e.g., streams and rivers), like the species in this study, stream flow is considered a master variable for the ecological integrity and structure of stream habitats (Poff et al., 1997). Stream flow has provided the evolutionary template to which fish life histories have diversified (Mims & Olden, 2012), and changes in flow magnitude and timing can be physiologically stressful, and alter habitat and distribution of food resources (Lytle & Poff, 2004; Poff & Ward, 1989). For example, high flows can be destructive by scouring the streambed, and low flow periods during drought can result in the drying of habitats needed for fish to carry out their life histories and exacerbate the negative effects of high water temperature on fish physiology.

Here, we quantified the effects of abiotic drivers on interspecific and spatial synchrony in bi‐monthly survival for two stream fish communities in geographically close but isolated sites using mark‐recapture methods. We first (1) tested for effects of environmental drivers on temporal variation in survival, then (2) estimated the relative degree of synchrony among species within a community (i.e., interspecific) and spatial synchrony among sites, and (3) determined the contribution of environmental covariates to driving interspecific and spatial synchrony. We predicted there could be spatial synchrony in survival between the two streams if populations responded similarly to environmental conditions and that ecologically similar species within each stream would be more synchronous with one another.

## 2 MATERIALS AND METHODS

### 2.1 Study sites

**FIGURE 2:**
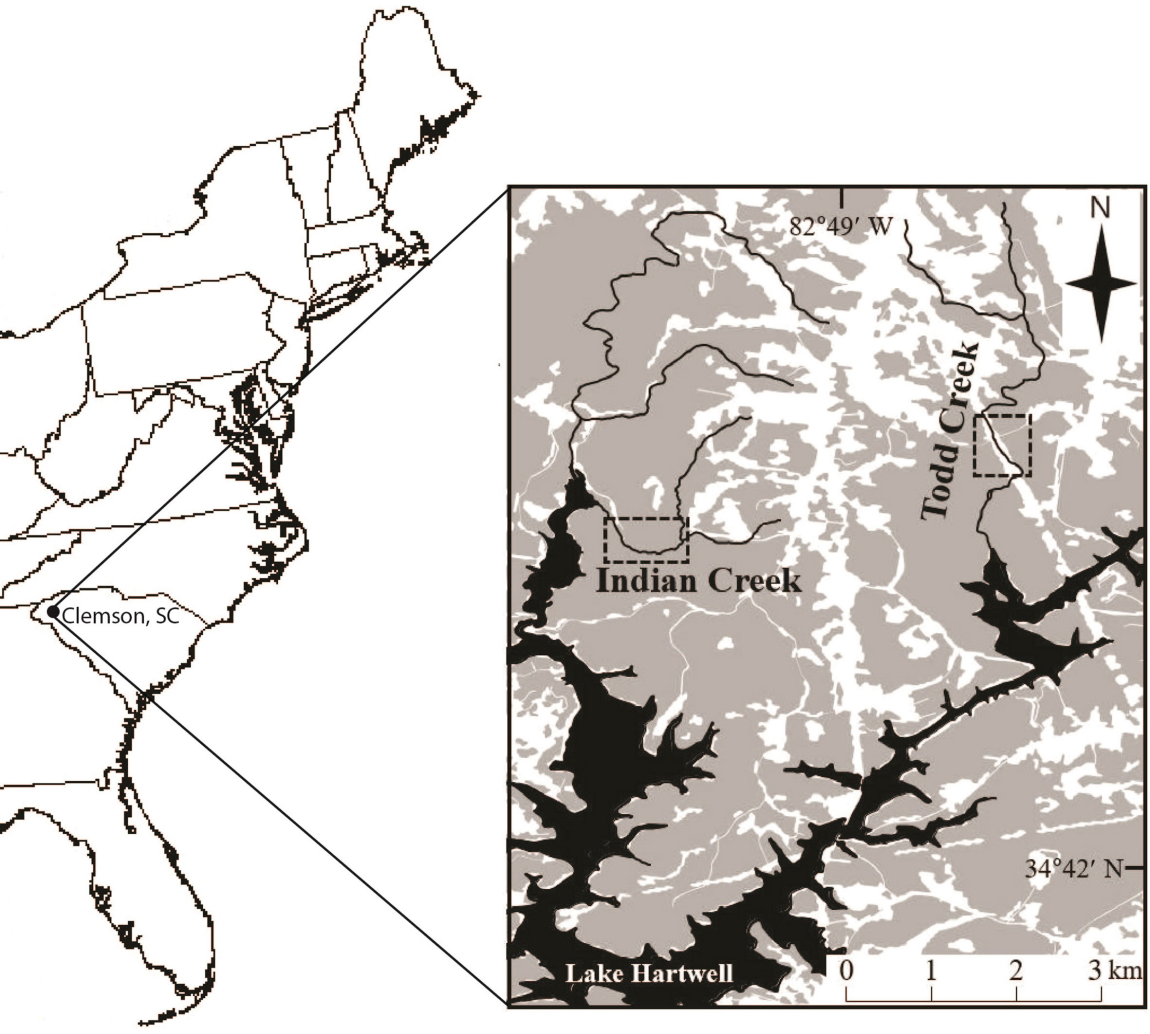
Study area map of Indian and Todd Creeks in Clemson, South Carolina, USA. Shaded gray areas represent forested land, black areas represent water bodies, and white areas are developed land and roads.

This study took place in Indian (34.741731° N, 82.849872° W) and Todd (34.749214° N, 82.813911° W) Creeks in the Clemson Experimental Forest, South Carolina, United States (Figure 2). Both are second‐order streams and similar in stream size, but Indian Creek (mean wetted width = 2.6 m; range = 0.7–6.2 m) has a forested riparian zone whereas Todd Creek (mean wetted width = 3.3 m; range = 1.4–7.0 m), located in a power‐line corridor, has an open canopy. Both streams are located in the same watershed and are approximately 3 km apart in Euclidean distance. Additionally, these two streams flow into a reservoir and are not directly connected to each other so dispersal between these two study sites is not possible (Figure 2). Target species included bluehead chub (*Nocomis leptocephalus*), creek chub (*Semotilus atromaculatus*), and striped jumprock (*Moxostoma rupicartes*). All three species have been classified as coolwater species in existing literature (Lyons et al., 2009; Myers et al., 2017), however, striped jumprock and creek chub have also been documented in both cool‐ and warm‐water streams (Nelson et al., 2021; Tracy et al., 2013) suggesting that they may have a wider thermal tolerance. Coolwater species tend to have a summer thermal range of 21–24°C (Beauchene et al., 2014; Lyons et al., 2009; McKenna Jr et al., 2010). These three species are more abundant in pool and run habitats, with bluehead chub and striped jumprock being more abundant in mid‐sized streams and are fluvial specialists (Freeman & Marcinek, 2006). Creek chubs are habitat generalists and are tolerant of a wide range of flows (Kanno & Vokoun, 2010). While little is published about the ecology of striped jumprock, they have a subterminal sucker mouth characteristic of their taxonomic family Catostomidae and attain the largest body size of the three species included in this study (Rohde et al., 2009). Bluehead chub and creek chub (Family Leuciscidae) are taxonomically and ecologically similar to one another in terms of diet (opportunistic generalists that consume aquatic plants and a diversity of invertebrates), and body morphology (Rohde et al., 2009). These three species also overlap in reproductive timing (March–June) where striped jumprock has been observed to spawn first, followed closely by creek chub and bluehead chub (Kim & Kanno, 2020).

Given the close proximity of our study sites, we predicted there could be spatial synchrony in survival between the two streams if species respond similar to climatic drivers. However, given that these sites are also isolated and lack dispersal, it is possible that asynchronous dynamics may also occur. For interspecific synchrony, if water temperature was an important driver of synchrony then we expected more synchronous responses among species due to this variable's importance to fish physiology (Kanno et al., 2023). In contrast, if flow metrics were more important to synchrony then we expected more asynchronous responses since our community harbors a combination of fluvial specialists and generalists.

### 2.2 Sampling methods

A bi‐monthly mark‐recapture survey was conducted in two streams from November 2015 to March 2018 for a total of 15 sampling occasions. Streams were divided into 20 m sections with 26 sections in Todd Creek (520 m) and 37 sections in Indian (740 m). Indian Creek had a longer study area to increase sample size given its lower fish density. These 20 m sections were sampled using pulsed‐DC backpack electrofishing with a Smith‐Root LR‐24 backpack unit (Smith‐Root, Inc.) and a Halltech HT‐2000 backpack unit (Halltech Aquatic Research, Inc.). A two‐pass depletion approach was used to increase recaptures. That is, each section was sampled twice, retaining fish captured in the first pass in a bucket when the section was sampled for the second time. On the first sampling occasion, all captured fish were identified to species and measured (mm) and weighed (g). Fish ≥60 mm in total length were then tagged with 8‐mm passive integrated transponder (PIT) tags (Oregon RFID; or Biomark). Detailed PIT tag incision protocols are described in Cary et al. (2017), and this previous study demonstrated that mortality and tag loss were negligible. On all subsequent occasions, the 20 m sections were sampled in a similar fashion where all captured fish were scanned with a handheld PIT tag reader (Avid PowerTracker 7), and previously tagged individuals (recaptures) were recorded, and non‐tagged fish were implanted with a PIT tag before they were returned to the section of capture alive. Field sampling was completed as quickly as possible to conform to the assumption of instantaneous sampling on each occasion (Kéry & Schaub, 2012). Sampling during each occasion was typically completed within a mean of 3 days (range = 1–10 days) in Indian Creek and within 4 days (range = 1–7 days) in Todd Creek. Intervals between sampling occasions were a mean of 61 days (range = 48–70 days) in both Indian and Todd Creeks. If species lack site fidelity, then survival estimates will be biased lower (referred to as apparent survival) than the true survival (Schaub & Royle, 2014). These fish species have low probabilities of emigrating from our study area (Terui et al., 2021) based on the short movement distances compared to the length of our study area. Therefore, we assumed that apparent survival was nearly equivalent to true survival. Temperature and water level loggers were deployed in each stream and measured hourly temperature and daily water level (HOBO Onset Computer Corp, Model U20L‐004). All fieldwork was conducted in accordance with protocols approved by the Clemson University Institutional Animal Care and Use Committee (IACUC Protocol Number 2014‐047 and 2017‐039).

### 2.3 Statistical analysis

We developed Cormack‐Jolly‐Seber (CJS) models for three separate statistical analyses to first (1) identify environmental drivers of survival, then (2) to quantify the magnitude of interspecific and spatial synchrony in survival, and finally (3) to estimate the contributions of the most supported environmental drivers to synchrony. Data for statistical analyses consisted of a capture history for each individual and occasion. Capture histories of all individuals (*i*), across sampling occasions (*t*) were created as a two‐dimensional array, $y_{i,t}$, where 1 s represent captures and 0 s for non‐captures for each individual and occasion.

### 2.4 Environmental drivers of survival

To identify environmental drivers of survival, we followed Kéry and Schaub (2012) and fit multispecies CJS models in each creek representing an ecological process (Equations 1 and 2) and observation process (Equation 3).

(1) $$z_{i,t+1}|z_{i,t}\sim \text{Bernoulli}\left(z_{i,t}\Phi_{i,t}\right),$$

(2) $$\text{logit}\left[\Phi_{i,t}\right]=\mu_{j\left(i\right)}+\beta_{j\left(i\right)}x_{t}$$

(3) $$y_{i,t}|z_{i,t}\sim \text{Bernoulli}\left(z_{i,t}p_{i,t}\right),$$

Where the inverse‐logit transformation of *μ*_*j*(*i*)_ represents the overall mean bi‐monthly survival rate for species *j* to which individual *i* belongs to, *β*_*j(i)*_ represents the effect size of covariate *x* for each species *j*(*i*). $\Phi_{i,t}$ refers to the bi‐monthly survival rate of individual *i* on a given occasion *t*. Survival was modeled conditional on the latent state of individual *i* on the immediately previous occasion (Equation 1), so that a dead individual (*z*_*i,t*_ = 0) remained dead and a live individual (*z*_*i,t*_ = 1) would survive to the next occasion with a probability of $\Phi_{i,t}$. Recapture probability was modeled to vary by occasion (*t*) and species (*j*). To account for different intervals between sampling occasions, we standardized survival to 60 days using $\Phi_{i,t}^{\frac{60}{n.\text{days}}}$, where *n*.days refers to the number of days between the median sampling day of one occasion and that of the following occasion. Todd and Indian Creek datasets were analyzed separately for this part of the analysis in case different covariates were affecting survival in each stream.

Five environmental covariates were considered to determine which covariates were most important to variation in bi‐monthly survival. These covariates included the maximum and mean daily water temperature, and the mean, max, and minimum daily water level for each interval between sampling occasions. Water level was used as a metric for stream flow. We summarized these covariates from the hourly water temperature and daily water level logger data. To be consistent with the water level time interval we calculated mean daily water temperature for each day of data. We considered both the mean and extremes (maximum and/or minimum) of these covariates to account for any influence of potential outliers since the magnitude of both temperature and flow metrics can influence fish demography (Little et al., 2020; Poff & Ward, 1989). We calculated pairwise Pearson's *r* correlation coefficients to check for correlation among environmental covariates, and covariates with strong correlation (Pearson's *r* > .50) were excluded from analyses (Dormann et al., 2012). In both streams, maximum temperature was highly correlated with mean temperature (Pearson's *r* > .90), and maximum and minimum water level was highly correlated with mean water level (Pearson's *r* > .50); therefore, we excluded the maximum temperature, and maximum and minimum water level covariates and retained two covariates: mean water temperature, and mean water level (Supporting Information S1, Figure S1). Water temperature and water level covariates were standardized by the mean divided by standard deviation prior to analysis. In the end, three models (a model for each covariate and a null model that was intercept only) were constructed using Equation 2 and compared for Todd and Indian Creek datasets (6 models in total). Models were ranked by Deviance Information Criterion (DIC), a Bayesian analogue to AIC (Spiegelhalter et al., 2002). DIC was calculated in the jagsUI package and the lowest DIC value represented the most supported model of those that were considered. We also checked for statistical significance of covariate effect sizes for each species and stream. Covariates to survival were considered significant if their 95% credible interval (CRI) did not overlap 0.

### 2.5 Estimating interspecific and spatial synchrony

Next, we combined capture histories for all species in both streams in a single model to quantify the magnitude of interspecific and spatial synchrony in survival using four random effects (Equation 4), where $\varepsilon 1_{t}$ was the random effect to estimate the temporal variance common to all species in both streams, $\varepsilon 2_{j\left(i\right),t}$ to estimate the temporal variance unique to each species, $\varepsilon 3_{s\left(i\right),t}$ to estimate the temporal variance unique to each stream (*s*(*i*)), and $\varepsilon 4_{j\left(i\right),t,s\left(i\right)}$ to estimate the temporal variance unique to each combination of species and stream. These four random effects were sampled from a normal distribution with a mean of 0 and variances,$\;\sigma_{t}^{2}$,$\;\sigma_{j}^{2}$, $\sigma_{s}^{2}$, $\sigma_{j,s}^{2}$, respectively.

(4) $$\text{logit}\left[\Phi_{i,t}\right]=\mu_{j\left(i\right),s\left(i\right)}+\varepsilon 1_{t}+\varepsilon 2_{j\left(i\right),t}+\varepsilon 3_{s\left(i\right),t}+\varepsilon 4_{j\left(i\right),t,s\left(i\right)}$$

We used two different approaches to quantify the amount of interspecific and spatial synchrony in our system. First, we calculated an intra‐class correlation coefficient (ICC), described in Grosbois et al. (2009) and Lahoz‐Monfort et al. (2011). ICC estimates represent the synchrony of a given species with the rest of the species for each stream community:

(5) $$\mathrm{ICC}=\frac{\sigma_{t}^{2}{+\sigma }_{j}^{2}\;{+\sigma }_{s}^{2}}{\sigma_{t}^{2}+\sigma_{j}^{2}+\sigma_{s}^{2}+\sigma_{j,s}^{2}}$$

ICCs quantify synchrony as a proportion of shared variance ($\sigma_{t}^{2}+\sigma_{j}^{2}+\sigma_{s}^{2}$) to total variance ($\sigma_{t}^{2}+\sigma_{j}^{2}+\sigma_{s}^{2}+\sigma_{j,s}^{2}$) for a given combination of species and stream (Equation 5). ICC values range from 0 to 1 and we assessed interspecific synchrony where values closer to 0 indicate low synchrony for a given species with the rest of the community, and values closer to 1 indicate high synchrony. We estimated a total of six ICC values, one for each species in each stream. To assess spatial synchrony, we compared these ICC values across streams, and if streams had different ICC results we interpreted this as potential evidence of spatial asynchrony. For our second approach we used Pearson's *r* correlations of bi‐monthly survival estimates from Equation 4 for each pairwise species comparison within and between streams to further assess interspecific and spatial synchrony. While ICC provides an estimate of synchrony, it characterizes the variation in a species' survival in comparison to the shared variance (i.e., with the rest of the community). We were also interested in synchrony among pairwise species comparisons both within and between these two communities to better understand which species had more similar and/or different survival patterns with one another. These two approaches taken together provided multiple lines of evidence for interpreting synchrony patterns.

### 2.6 Contributions of environmental drivers to synchrony

To estimate the contributions of environmental drivers to synchrony, the most supported environmental covariate from Equation 2 identified by model selection (i.e., the model with the lowest DIC value) was then incorporated into the random effects model described in Equation 4. This new model with both random effects and the environmental covariate (${\mathrm{\beta x}}_{t}$) is described in Equation 6.

(6) $$\text{logit}\left[\Phi_{i,t}\right]=\mu_{j\left(i\right),s\left(i\right)}+{\mathrm{\beta x}}_{t}+\varepsilon 1_{t}+\varepsilon 2_{j\left(i\right),t}+\varepsilon 3_{s\left(i\right),t}+\varepsilon 4_{j\left(i\right),t,s\left(i\right)}$$

We averaged this covariate ($x_{t}$) across both streams for each occasion since the same covariate affected survival in both streams (see results section). We estimated the synchronous variance explained by the top covariate ($\sigma_{\mathrm{cov}}^{2}$) by calculating the variance in a vector of the product of the covariate effect size (β) multiplied by each occasion's covariate value ($x_{t}$) following the methods detailed in Nakagawa and Schielzeth (2013) and Ghislain et al. (2022). We then calculated the proportion of synchronous variation accounted for by the covariate using the overall variance term ($\sigma_{t}^{2}$) estimated in Equation 5 and the temperature variance term ($\sigma_{\mathrm{cov}}^{2}$) described above as $\frac{\sigma_{\mathrm{cov}}^{2}}{\sigma_{\mathrm{cov}}^{2}+\sigma_{t}^{2}}$. We also compared DIC values between the two models from Equation 4: Model $\Phi \left(\varepsilon 1+\varepsilon 2+\varepsilon 3+\varepsilon 4\;\right)$ and Equation 6: Model $\Phi \left(\mathrm{cov}+\varepsilon 1+\varepsilon 2+\varepsilon 3+\varepsilon 4\right)$ as further evidence for whether environmental covariates influenced synchrony. Finally, to assess model fit, we also ran an intercept only model (i.e., without random effects or covariates) and compared the DIC of this intercept model to the DIC values of Equations 4 and 6 models. If the intercept model had a higher DIC value than Equations 4 and 6, then we interpreted that the random effects and covariates improved model fit.

### 2.7 Model fitting

CJS models were fit with the jagsUI package (Kellner, 2014) from program R (R Core Development Team, 2022). Posterior distributions of model parameters were estimated using diffuse priors (Table S1) and by taking every 10th sample from 250,000 iterations after discarding 10,000 burn‐in iterations for three Markov Monte Carlo chains for the Equation 2 models. Synchrony models (Equations 4 and 6) were run longer with 600,000 iterations and 100,000 burn‐in. Model convergence was checked by visually examining plots of the Markov chains for adequate mixture and ensuring that the potential scale reduction factor value was less than 1.1 for all model parameters (Gelman & Hill, 2007). Example JAGS code for survival models can be found in Supporting Information S3.

## 3 RESULTS

### 3.1 Species' survival summary

**FIGURE 3:**
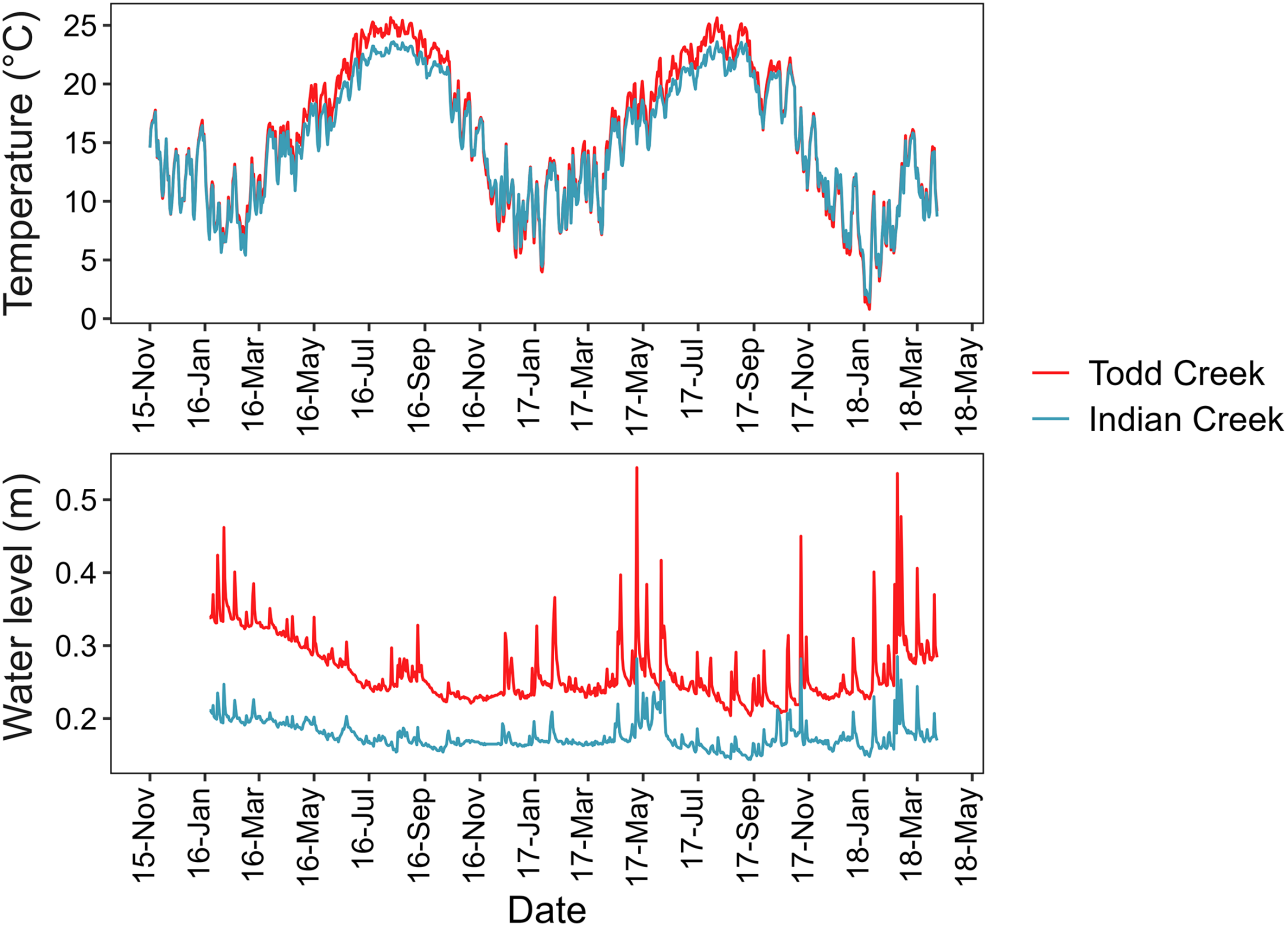
Mean daily temperature (°C) from November 2016 to March 2018 and water level (m) from January 2016 to March 2018 for Todd and Indian Creeks.

Over the 28‐month study period, 1337 unique individuals ≥60 mm were tagged in Indian Creek and 4442 in Todd Creek (Table S2). Creek chub had the greatest number of individuals in Indian Creek (50% of total tagged) whereas bluehead chub made up the majority of tagged individuals in Todd Creek (81% of total tagged). More creek chub (664 vs. 195) were tagged in Indian Creek and more striped jumprock and bluehead chub (639 vs. 244; 3608 vs. 429) were tagged in Todd Creek. Average daily temperature was similar for both sites (Pearson's *r* = .99, *p* < .05), (Indian Creek, mean = 15.2°C, range = 1.4–23.6°C; Todd Creek, mean = 15.8°C, range = 0.7–25.6°C) (Figure 3), but differences in stream temperature were greatest during July‐Sept 2016 where Todd Creek was 2.1°C warmer compared to Indian Creek. Water level was correlated over time between streams (Pearson's *r* = .81, *p* < .05) but we observed some differences where average water level was lower in Indian Creek (mean = 17 cm; range = 14–28 cm) compared to Todd Creek (mean = 26 cm; range = 20–54 cm; Figure 3). Todd Creek had a greater magnitude in peak flow after winter precipitation events relative to fall and summer.

**FIGURE 4:**
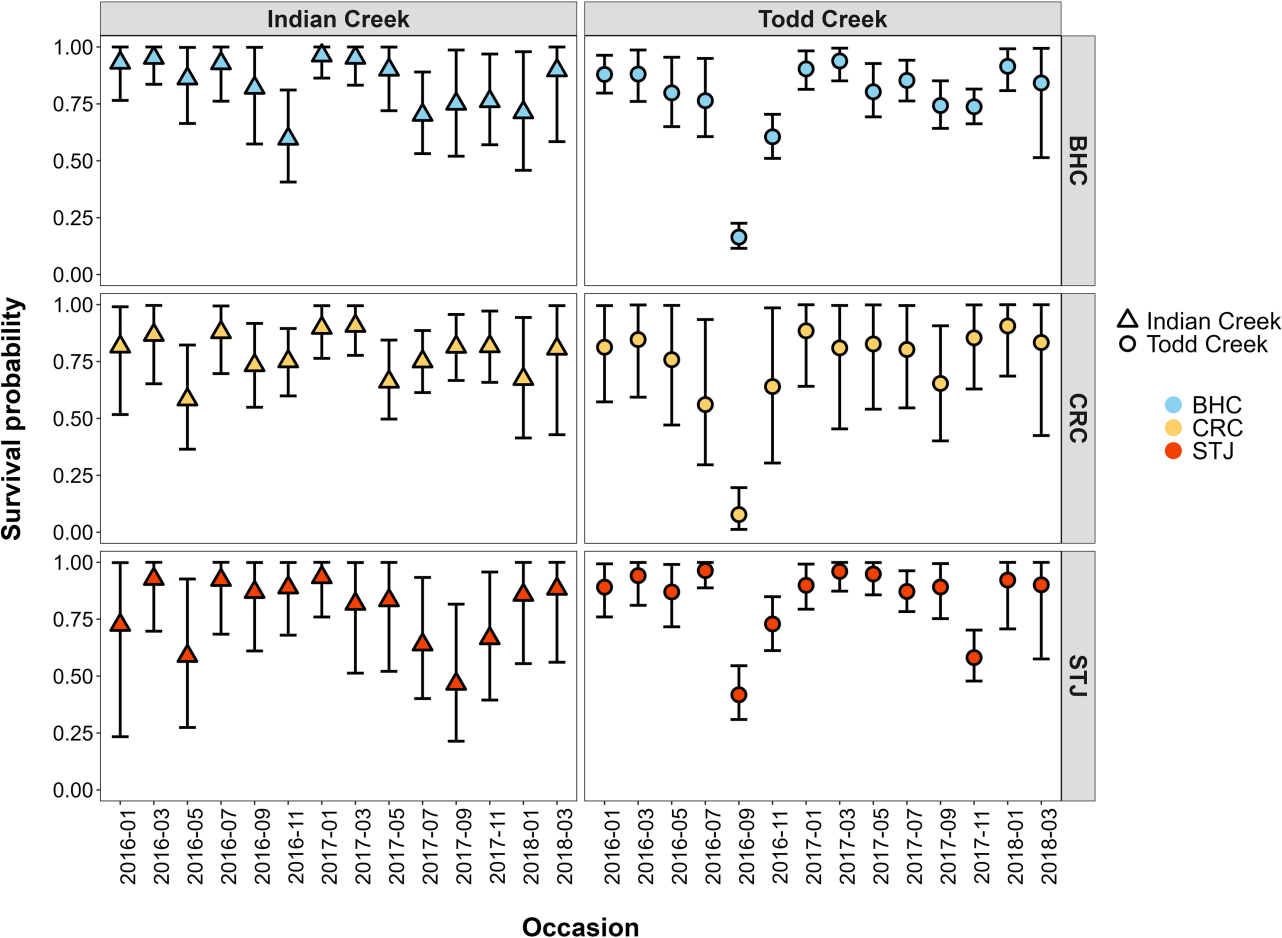
Estimated apparent bi‐monthly survival probability for the species present in Indian and Todd Creeks from model $\Phi \left(\varepsilon 1+\varepsilon 2+\varepsilon 3+\varepsilon 4\right)$. Where “2016‐01” represents the first bi‐monthly occasion from November 2015 to January 2016. Point estimates are the mean of the MCMC posterior distribution samples for survival of each species. Vertical bars show 95% credible intervals.

Recapture probabilities varied across species and occasions (range = 0.18–0.53, Figure S2). Survival differed greatly by time, stream, and species (range = 0.07–0.96), where survival was lower in late summer (July–September; Figure 4). Between July and September 2016, fish survival in Todd Creek was much lower than Indian Creek where we observed a survival range of 0.07–0.48 in Todd Creek compared to a range of 0.73–0.86 in Indian Creek for the same occasion.

### 3.2 Environmental drivers of survival

Model selection results showed that mean water temperature negatively affected survival in each stream (Table S3). In Indian Creek, survival decreased with mean water temperature for bluehead chub (effect size = −0.54, 95% CRI = −0.72 to −0.34), creek chub (effect size = −0.37; 95% CRI = −0.56 to −0.17), and striped jumprock (effect size = −0.49; 95% CRI = −0.86 to −0.11; Table S4). Todd Creek had larger significant effect sizes of mean water temperature on survival relative to Indian Creek for bluehead chub and creek chub. Survival decreased with mean temperature for bluehead chub (effect size = −0.82, 95% CRI = −0.98 to −0.62) and creek chub (effect size = −0.71, 95% CRI = −1.20 to −0.10) present in Todd Creek, and was non‐significant (95% CRI overlapped 0) for striped jumprock (Table S4). Mean water level was less supported by model selection (Table S3), and statistically significant for only bluehead chub in Indian (effect size = 0.47, 95% CRI = 0.22 to 0.71) and Todd Creeks (effect size = 0.19, 95% CRI = 0.04 to 0.37) where survival increased with higher mean water level. Mean water level was non‐significant for creek chub and striped jumprock in both streams.

### 3.3 Interspecific and spatial synchrony

Intra‐class and Pearson's correlation coefficients revealed that the two streams had different synchrony patterns. Intra‐class correlation coefficients (ICC) ranged from 0.58 to 0.74 (mean ICC = 0.63) in Indian Creek and 0.66–0.84 (mean ICC = 0.74) in Todd, suggesting lower interspecific synchrony among species in Indian Creek compared to those in Todd Creek (Table 1). However, ICC 95% credible intervals were wide, particularly in Indian Creek (range 0.18–0.96). Pearson's correlation coefficients showed similar patterns to ICC results where Todd Creek had larger interspecific correlation values than Indian Creek. None of the interspecific correlation comparisons in Indian Creek were statistically significant (Pearson's *r* range = 0.30–0.41, *p* > .05; Table 2, Figure S3). Pearson's correlations for species pairwise comparisons in Todd Creek were all significant with a range of 0.67–0.93 (Table 2, Figure S4). When examining spatial synchrony (i.e., comparing species across streams), all three possible species pairs had non‐significant Pearson's *r* correlations (range = 0.04–0.30, Table 2) and illustrated spatial asynchrony in bi‐monthly survival across the two streams (Figure S5). Taken together, our results most closely aligned with a scenario with interspecific synchrony and spatial asynchrony (Figure 1b).

**TABLE 1:** Intra‐class correlation coefficients (ICC, 95% CRI) for each species in each stream for Equation 4, Model $\Phi \left(\varepsilon 1+\varepsilon 2+\varepsilon 3+\varepsilon 4\right)$, and derived covariate variance ($\sigma_{\mathrm{cov}}^{2}$) for Equation 6, Model $\Phi \left(\mathrm{cov}+\varepsilon 1+\varepsilon 2+\varepsilon 3+\varepsilon 4\right)$.

	$\sigma_{t}^{2}$	$\sigma_{s}^{2}$	$\sigma_{j}^{2}$	$\sigma_{j,s}^{2}$	ICC
Within & among stream synchrony: Model $\Phi \;\left(\varepsilon 1+\varepsilon 2+\varepsilon 3+\varepsilon 4\;\right)$	0.60	0.64 ($\sigma_{\text{Indian}}^{2}$)	0.42 ($\sigma_{\mathrm{BHC}}^{2}$)	1.33 ($\sigma_{\mathrm{BHC},\text{Indian}}^{2}$)	0.58 (0.18, 0.96)
		1.08 ($\sigma_{\text{Todd}}^{2}$)	0.44 ($\sigma_{\mathrm{CRC}}^{2}$)	0.60 ($\sigma_{\mathrm{CRC},\text{Indian}}^{2}$)	0.74 (0.40, 0.98)
			0.80 ($\sigma_{\mathrm{STJ}}^{2}$)	1.50 ($\sigma_{\mathrm{STJ},\text{Indian}}^{2}$)	0.59 (0.24, 0.96)
				0.42 ($\sigma_{\mathrm{BHC},\text{Todd}}^{2}$)	0.84 (0.57, 0.99)
				1.28 ($\sigma_{\mathrm{CRC},\text{Todd}}^{2}$)	0.66 (0.28, 0.98)
				0.98 ($\sigma_{\mathrm{STJ},\text{Todd}}^{2}$)	0.73 (0.42, 0.98)
Contribution of covariate to synchrony: Model $\Phi \left(\mathrm{cov}+\varepsilon 1+\varepsilon 2+\varepsilon 3+\varepsilon 4\right)$	0.41	0.79 ($\sigma_{\text{Indian}}^{2}$)	0.33 ($\sigma_{\mathrm{BHC}}^{2}$)	1.14 ($\sigma_{\mathrm{BHC},\text{Indian}}^{2}$)	
		0.81 ($\sigma_{\text{Todd}}^{2}$)	0.49 ($\sigma_{\mathrm{CRC}}^{2}$)	0.89 ($\sigma_{\mathrm{CRC},\;\text{Indian}}^{2}$)	
			0.78 ($\sigma_{\mathrm{STJ}}^{2}$)	1.37 ($\sigma_{\mathrm{STJ},\text{Indian}}^{2}$)	
				0.36 ($\sigma_{\mathrm{BHC},\text{Todd}}^{2}$)	
				1.09 ($\sigma_{\mathrm{CRC},\text{Todd}}^{2}$)	
				1.07 ($\sigma_{\mathrm{STJ},\text{Todd}}^{2}$)	
Derived covariate variance: $\sigma_{\mathrm{cov}}^{2}$ = 0.60					

*Note*: Estimated random effect variances (𝜎^2^) for each random effect term are reported for both Equations 4 and 6 models.

**TABLE 2:** Pearson's *r* correlations between bi‐monthly survival estimates for bluehead chub (BHC), creek chub (CRC), striped jumprock (STJ) for pairwise comparisons among species in (a) Indian Creek, (b) Todd Creek, and (c) between streams.

Species pair	Correlation	*p*‐value	Species pair	Correlation	*p*‐value
(a) Indian creek			(b) Todd creek		
BHC versus CRC	0.41	.14	BHC versus CRC	0.93	**<.005**
BHC versus STJ	0.32	.26	BHC versus STJ	0.85	**<.005**
CRC versus STJ	0.30	.29	CRC versus STJ	0.67	**.008**
(c) Between sites					
BHC_Indian_ versus BHC_Todd_	0.30	.28			
CRC_Indian_ versus CRC_Todd_	0.09	.75			
STJ_Indian_ versus STJ_Todd_	0.04	.88			

*Note*: Survival estimates from model $\Phi \;\left(\varepsilon 1+\varepsilon 2+\varepsilon 3+\varepsilon 4\;\right)$. Significant correlations (*p* < .05) are in bold.

### 3.4 Contribution of environmental covariates to synchrony

We found support for mean temperature influencing synchrony across both streams. The proportion of synchronous variation accounted for by mean temperature was 0.49 (95% CRI = 0.04–0.95). Differences in DIC values between the two synchrony CJS models (Equations 4 and 6) provided additional evidence mean temperature contributed to synchrony in both streams. The synchrony model with mean temperature (Equation 6) had a lower DIC (DIC = 23,080) compared with the model without covariates (Equation 4; DIC = 24,742) and the intercept model (DIC = 30,465) which suggests that including the four random effects and temperature covariate improved model fit. Synchrony model output (Equations 4 and 6) for all monitored parameters can be found in Supporting Information S2 (Table S5 and Table S6).

## 4 DISCUSSION

Understanding the degree of demographic variation has important implications for the conservation and persistence of species and populations. Temporal variation in survival in these stream fish communities was best characterized by patterns of interspecific synchrony and spatial asynchrony. Specifically, synchrony was significant in all pairwise species comparisons in Todd Creek, and spatial synchrony was weak (i.e., non‐significant) despite geographic proximity of the two study streams (c.a. 3 km apart). Notably, the degree of interspecific synchrony varied between the two streams due in part to differences in summer water temperature regimes where habitat variation interacted with regional climate drivers resulting in spatial asynchrony in survival. This suggests that fine‐scale habitat variation can aid in buffering against negative effects of stressful environmental conditions like warming water temperatures. Interspecific and spatial synchrony are rarely investigated simultaneously and this work provides important knowledge on how variation in environmental conditions can synchronize local communities as well as interact with local habitat to generate spatial variation in demography of the same species.

Bi‐monthly survival was spatially asynchronous between our two study streams and these results contrasted with what is typically expected of spatial relationships of synchrony, where geographically close populations can exhibit synchronous dynamics (Lundberg et al., 2000; Ranta et al., 1995). However, we do note that while our sites were geographically close, they were also isolated without possible dispersal. Asynchrony among geographically close but isolated sites can arise due to differences in population characteristics like local adaptation to habitat variation (Hilborn et al., 2003; Rogers & Schindler, 2008). Despite close geographical proximity and similar stream size and trends in patterns of water temperature and level over time, the two study streams differed in habitat characteristics, particularly riparian deforestation in Todd Creek compared to the well forested Indian Creek. This lack of riparian cover in Todd Creek likely resulted in higher summer water temperatures (Danehy et al., 2005), and different survival patterns between the two streams. Water temperature negatively affected species' survival in both streams, but the magnitude of this effect was much stronger in Todd relative to Indian. Notably, survival between July and September 2016 differed markedly between the two streams, and water temperatures differed most in summer 2016 (~2°C difference) relative to 2017 (~1°C difference). Our data suggest climate drivers such as air temperature interacted with local‐scale conditions (i.e., riparian condition) to generate spatial asynchrony in demography, observed similarly in other taxa such as amphibians (Cayuela et al., 2016). Recent advances in macrosystems ecology have identified how multiscale systems, such as riverine networks, can drive the community patterns and processes (Heffernan et al., 2014). Riverine habitat characteristics such as substrate size and channel morphology change from upstream to downstream (Vannote et al., 1980), and this longitudinal shift is attributed to provide a broad‐scale template of spatial heterogeneity on which cross‐scale interactions between local (e.g., habitat) and regional drivers (e.g., climate) occur in riverine networks (Heffernan et al., 2014) to generate biocomplexity and influence population dynamics. Our study shows that fine‐scale interactions also occur due to differences in stream habitat quality and we think this is the more likely explanation for observed differences in survival patterns between the two streams rather than local adaptation.

The degree of interspecific synchrony also differed between the two streams. It is particularly notable that high synchrony in Todd Creek was created by high summer mortality rates across species, particularly in 2016 when summer temperatures differed more between the two streams than 2017. This result shows that climate variables like temperature was magnified (Stenseth et al., 2004) by an anthropogenic habitat alteration (i.e., riparian deforestation). Stream temperature is a major factor that structures fish communities (Jackson et al., 2001) and communities may shift readily along a narrow thermal gradient. Coolwater communities, to which some of our study species belong, occupy streams with summer temperatures ranging up to 21–24°C (Beauchene et al., 2014; Lyons et al., 2009; McKenna Jr et al., 2010). Mean daily temperatures between July and September 2016 were just at this upper threshold in Todd Creek (24°C) and approached it in Indian Creek (22°C). Although seemingly small, the difference in summer temperatures between the two streams would be sufficient to trigger spatially heterogeneous patterns of fish survival. Finally, water level was less important to variation in survival over time except for bluehead chub, which had higher survival during periods of higher flows, likely due to this species being a fluvial specialist.

Studies have also linked interspecific synchrony to the biological characteristics of species, where functionally similar (e.g., life history strategies or morphology) species exhibit similar population dynamics (Kanno et al., 2023; Rocha et al., 2011; Tedesco & Hugueny, 2006). The moderate to high interspecific synchrony observed in our study streams could be due to the sensitivity of fish physiology to warmer water temperatures. Additionally, taxonomic similarities may account for the high synchrony observed between bluehead chub and creek chub. Perhaps we would have observed more interspecific asynchrony if we had higher ecological trait diversity in our sites. Furthermore, the southeastern United States has higher proportions of small‐bodied species with opportunistic life history traits (e.g., short generation time, high reproductive effort; Winemiller, 2005). While this region experiences high community turnover due to having more dynamic habitats (e.g., a more variable flow regime), opportunistic life history traits allows these species to quickly recover (Grossman et al., 1990; Mims & Olden, 2012). In addition, our study duration was short (~28 months) and community turnover (thus more asynchronous demography) may have been revealed over a longer temporal extent.

Identifying key environmental drivers of low survival within the annual cycle provides important knowledge for the conservation of species. A likely consequence of global change is an increase in the occurrence of extreme climatic events (Easterling et al., 2000). We observed that physiologically stressful events, such as high water temperatures, have the potential to synchronize whole communities. Ecological theory predicts that communities exist in a “loose equilibrium” state meaning while stochastic environmental events can alter population vital rates or trends, they will eventually return back to average condition (Matthews et al., 2013; Matthews & Marsh‐Matthews, 2016). While populations may tolerate one event like this, sustained and frequent extreme events (within and/or among years), could negatively impact the population dynamics and persistence of short‐lived species', like those included in our study and may put these communities at risk of losing their resiliency to recover back to this average condition. Spatial asynchrony in survival among populations of the same species can decrease extinction risk (e.g., meta‐population persistence) and asynchrony among species within communities can facilitate species coexistence and relax interspecific competition over food resources or space (Siepielski & McPeek, 2010). Furthermore, our data suggest that spatial asynchrony likely occurred due to fine‐scale habitat differences (i.e., riparian cover in Indian Creek) that buffered against high water temperatures. This shows that some sites may be more resilient in the face of environmental change relative to others and this modeling framework can aid in the identification of target sites for conservation.

Our modeling framework is widely applicable to animal communities distributed in a landscape. Although we used an intensive mark‐recapture approach to infer interspecific and spatial synchrony in survival, other types of data such as abundance could also be used to partition temporal variation within and among communities using a set of random effects. Such an approach would more readily make inferences across a greater number of species and sites but at the expense of detailed demographic information such as survival estimates. Additionally, characterizing population trends over time can be challenging due to temporal and spatial stochasticity (Pregler et al., 2019) and our results have important implications for monitoring designs. For example, understanding the degree of correlation in species/population trends over time is important in deciding how many sites may be needed in monitoring surveys to accurately characterize spatial heterogeneity. Overall, our data demonstrate sensitivity of aquatic ectotherms to warming and a unique insight gained by conducting mark‐recapture studies at fine temporal scales (i.e., bi‐monthly). Annual sampling, which is more typical in mark‐recapture studies, cannot reveal seasonal patterns in demography, and this bottleneck period would have otherwise been missed. More research is needed on patterns and drivers of demographic synchrony within and among animal communities, and filling this knowledge gap is paramount to projecting community shifts and informing biodiversity conservation.

## AUTHOR CONTRIBUTIONS

**Kasey C. Pregler:** Conceptualization (lead); data curation (lead); formal analysis (lead); investigation (lead); methodology (lead); writing – original draft (lead); writing – review and editing (lead). **Xinyi Lu:** Formal analysis (supporting); writing – review and editing (supporting). **George P. Valentine:** Formal analysis (supporting); writing – review and editing (supporting). **Seoghyun Kim:** Data curation (equal); writing – review and editing (supporting). **Yoichiro Kanno:** Conceptualization (supporting); data curation (equal); formal analysis (supporting); writing – review and editing (equal).

## CONFLICT OF INTEREST STATEMENT

The authors declare that we have no conflicts of interest.

## Supporting information


Data S1

## Data Availability

Data are available upon request and model code is available in Supporting Information.

## References

[ece310700-bib-0001] Adler, G. H. (1994). Tropical forest fragmentation and isolation promote asynchrony among populations of a frugivorous rodent. Journal of Animal Ecology, 63, 903–911.

[ece310700-bib-0002] Beauchene, M. , Becker, M. , Bellucci, C. J. , Hagstrom, N. , & Kanno, Y. (2014). Summer thermal thresholds of fish community transitions in Connecticut streams. North American Journal of Fisheries Management, 34, 119–131. 10.1080/02755947.2013.855280

[ece310700-bib-0003] Beisner, B. E. , Peres‐Neto, R. , Lindstrom, E. S. , Barnett, A. , & Lorena Longhi, M. (2006). The role of environmental and spatial processes in structuring lake communities from bacteria to fish. Ecology, 87, 2985–2991. 10.1890/0012-9658(2006)87[2985:TROEAS]2.0.CO;2 17249222

[ece310700-bib-0004] Bino, G. , Brandis, K. , Kingsford, R. T. , & Porter, J. (2020). Waterbird synchrony across Australia's highly variable dryland rivers – Risks and opportunities for conservation. Biological Conservation, 243, 108497. 10.1016/j.biocon.2020.108497

[ece310700-bib-0005] Bond, N. R. , Balcombe, S. R. , Crook, D. A. , Marshall, J. C. , Menke, N. , & Lobegeiger, J. S. (2015). Fish population persistence in hydrologically variable landscapes. Ecological Applications, 25, 901–913. 10.1890/14-1618.1 26465032

[ece310700-bib-0006] Bouchard, C. , Buoro, M. , Lebot, C. , & Carlson, S. M. (2022). Synchrony in population dynamics of juvenile Atlantic salmon: Analyzing spatiotemporal variation and the influence of river flow and demography. Canadian Journal of Fisheries and Aquatic Sciences, 79, 782–794. 10.1139/cjfas-2021-0017

[ece310700-bib-0007] Brown, J. H. , & Kodric‐Brown, A. (1977). Turnover rates in insular biogeography: Effect of immigration on extinction. Ecology, 58, 445–449. 10.2307/1935620

[ece310700-bib-0008] Cary, J. B. , Holbrook, J. L. , Reed, M. E. , Austin, T. B. , Steffensen, M. S. , Kim, S. , Pregler, K. C. , & Kanno, Y. (2017). Survival of upper piedmont stream fishes implanted with 8‐mm passive integrated transponder tags. Transactions of the American Fisheries Society, 146, 1223–1232. 10.1080/00028487.2017.1370015

[ece310700-bib-0009] Cayuela, H. , Arsovski, D. , Thirion, J. M. , Bonnaire, E. , Pichenot, J. , Boitaud, S. , Brison, A. L. , Miaud, C. , Joly, P. , & Besnard, A. (2016). Demographic responses to weather fluctuations are context dependent in a long‐lived amphibian. Global Change Biology, 22, 2676–2687. 10.1111/gcb.13290 27002592

[ece310700-bib-0010] Chevalier, M. , Laffaille, P. , & Grenouillet, G. (2014). Spatial synchrony in stream fish populations: Influence of species traits. Ecography, 37, 960–968. 10.1111/ecog.00662

[ece310700-bib-0011] Cline, T. J. , Muhlfeld, C. C. , Kovach, R. , Al‐Chokhachy, R. , Schmetterling, D. , Whited, D. , & Lynch, A. J. (2022). Socioeconomic resilience to climatic extremes in a freshwater fishery. Science Advances, 8, eabn1396. 10.1126/sciadv.abn1396 36070376 PMC9451147

[ece310700-bib-0012] Danehy, R. J. , Colson, C. G. , Parrett, K. M. , & Duke, S. D. (2005). Patterns and sources of thermal heterogeneity in small mountain streams within a forested setting. Forest Ecology and Management, 208, 287–302. 10.1016/j.foreco.2004.12.006

[ece310700-bib-0013] Dibner, R. R. , DeMarche, M. L. , Louthan, A. M. , & Doak, D. F. (2019). Multiple mechanisms confer stability to isolated populations of a rare endemic plant. Ecological Monographs, 89, e01360. 10.1002/ecm.1360

[ece310700-bib-0014] Dormann, C. F. , Elith, J. , Bacher, S. , Buchmann, C. , Carl, G. , Carré, G. , García Marquéz, J. R. , Gruber, B. , Lafourcade, B. , Leitão, P. J. , Münkemüller, T. , McClean, C. , Osborne, P. E. , Reineking, B. , Schröder, B. , Skidmore, A. K. , Zurell, D. , & Lautenbach, S. (2012). Collinearity: A review of methods to deal with it and a simulation study evaluating their performance. Ecography, 36, 27–46. 10.1111/j.1600-0587.2012.07348.x

[ece310700-bib-0015] Easterling, D. R. , Evans, J. L. , Groisman, P. Y. , Karl, T. R. , Kunkel, K. E. , & Ambenje, P. (2000). Observed variability and trends in extreme climate events: A brief review. Bulletin of the American Meteorological Society, 81, 417–425. 10.1175/1520-0477(2000)081<0417:OVATIE>2.3.CO;2

[ece310700-bib-0016] Elmqvist, T. , Folke, C. , Nyström, M. , Peterson, G. , Bengtsson, J. , Walker, B. , & Norberg, J. (2003). Response diversity, ecosystem change, and resilience. Frontiers in Ecology and the Environment, 1, 488–494. 10.1890/1540-9295(2003)001[0488:RDECAR]2.0.CO;2

[ece310700-bib-0017] Freeman, M. C. , & Marcinek, P. A. (2006). Fish assemblage responses to water withdrawals and water supply reservoirs in Piedmont streams. Environmental Management, 38, 435–450. 10.1007/s00267-005-0169-3 16688514

[ece310700-bib-0018] Gelman, A. , & Hill, J. (2007). Data analysis using regression and multilevel/hierarchical models. Cambridge University Press.

[ece310700-bib-0019] Ghislain, M. , Bonnet, T. , Godeau, U. , Dehorter, O. , Gimenez, O. , & Henry, P. (2022). Synchrony in adult survival is remarkably strong among temperate songbirds across France. *EcoEvoRxiv*. Preprint. Version 3 Preprint published on 2022‐05‐03, 01:41 and revised on 2022‐05‐05, 22:18. 10.32942/osf.io/cyeb7

[ece310700-bib-0020] Grosbois, V. , Harris, M. P. , Anker‐Nilssen, T. , McCleery, R. H. , Shaw, D. N. , Morgan, B. J. T. , & Gimenez, O. (2009). Modeling survival at multi‐population scales using mark‐recapture data. Ecology, 90, 2922–2932. 10.1890/08-1657.1 19886500

[ece310700-bib-0021] Grossman, G. D. , Dowd, J. F. , & Crawford, M. (1990). Assemblage stability in stream fishes: A review. Environmental Management, 14, 661–671. 10.1007/BF02394716

[ece310700-bib-0022] Haddad, N. M. , Holyoak, M. , Mata, T. M. , Davies, K. F. , Melbourne, B. A. , & Preston, K. (2008). Species' traits predict the effects of disturbance and productivity on diversity. Ecology Letters, 11, 348–356. 10.1111/j.1461-0248.2007.01149.x 18201199

[ece310700-bib-0023] Hammond, M. , Loreau, M. , de Mazancourt, C. , & Kolasa, J. (2020). Disentangling local, metapopulation, and cross‐community sources of stabilization and asynchrony in metacommunities. Ecosphere, 11, e03078. 10.1002/ecs2.3078 33324497 PMC7116476

[ece310700-bib-0024] Hansen, B. B. , Grøton, V. , Aanes, R. , Sæther, B. E. , Stien, A. , Fuglei, E. , Ims, R. A. , Yoccoz, N. G. , & Pedersen, A. Ø. (2013). Climate events synchronize dynamics of a resident vertebrate community in the high arctic. Science, 339, 313–315. 10.1126/science.1226766 23329044

[ece310700-bib-0025] Heffernan, J. B. , Soranno, P. A. , Angilletta, M. J., Jr. , Buckley, L. B. , Gruner, D. S. , Keitt, T. H. , Kellner, J. R. , Kominoski, J. S. , Rocha, A. V. , Xiao, J. , Harms, T. K. , Goring, S. J. , Koenig, L. W. , McDowell, W. H. , Powell, H. , Richardson, A. D. , Stow, C. A. , Vargas, R. , & Weathers, K. C. (2014). Macrosystems ecology: Understanding ecological patterns and processes at continental scales. Frontiers in Ecology and the Environment, 12, 5–14. 10.1890/130017

[ece310700-bib-0026] Hilborn, R. , Quinn, T. P. , Schindler, D. E. , & Rogers, D. E. (2003). Biocomplexity and fisheries sustainability. Proceedings of the National Academy of Sciences of the United States of America, 100, 6564–6568. 10.1073/pnas.1037274100 12743372 PMC164486

[ece310700-bib-0027] Hinks, A. E. , Cole, E. F. , Daniels, K. J. , Wilkin, T. A. , Nakagawa, S. , & Sheldon, B. C. (2015). Scale‐dependent phenological synchrony between songbirds and their caterpillar food source. The American Naturalist, 186, 84–97. 10.1086/681572 26098341

[ece310700-bib-0028] Hordley, L. A. , Gillings, S. , Petchey, O. L. , Tobias, J. A. , & Oliver, T. H. (2021). Diversity of response and effect traits provides complementary information about avian community dynamics linked to ecological function. Functional Ecology, 35, 1938–1950. 10.1111/1365-2435.13865

[ece310700-bib-0029] Huitu, O. , Norrdahl, K. , & Korpimäki, E. (2004). Competition, predation, and interspecific synchrony in cyclic small mammal communities. Ecography, 27, 197–206. 10.1111/j.0906-7590.2003.03684.x

[ece310700-bib-0030] Ives, A. R. , Dennis, B. , Cottingham, K. L. , & Carpenter, S. R. (2003). Estimating community stability and ecological interactions from time‐series data. Ecological Monographs, 73, 301–330. 10.1890/0012-9615(2003)073[0301:ECSAEI]2.0.CO;2

[ece310700-bib-0031] Jackson, D. A. , Peres‐Neto, P. R. , & Olden, J. D. (2001). What controls who is where in freshwater fish communities – The roles of biotic, abiotic, and spatial factors. Canadian Journal of Fisheries and Aquatic Sciences, 58, 157–170. 10.1139/f00-239

[ece310700-bib-0089] Kanno, Y. , Kim, S. , & Pregler, K. C. (2023). Sub‐seasonal correlation between growth and survival in three sympatric aquatic ectotherms. Oikos, e09685. 10.1111/oik.09685

[ece310700-bib-0032] Kanno, Y. , & Vokoun, J. (2010). Evaluating effects of water withdrawals and impoundments on fish assemblages in southern New England streams, USA. Fisheries Management and Ecology, 17, 272–283. 10.1111/j.1365-2400.2009.00724.x

[ece310700-bib-0033] Kellner, K. (2014). jagsUI: Run JAGS (specifically, libjags) from R; an alternative user interface for rjags. R package version 3.5.2. 2019 .

[ece310700-bib-0034] Kendall, B. E. , Bjørnstad, O. N. , Bascompte, J. , Keitt, T. H. , & Fagan, W. F. (2000). Dispersal, environmental correlation, and spatial synchrony in population dynamics. The American Naturalist, 155, 628–636. 10.1086/303350 10777435

[ece310700-bib-0035] Kéry, M. , & Schaub, M. (2012). Bayesian population analysis using WinBUGS: A hierarchical perspective. Academic Press.

[ece310700-bib-0036] Kim, S. , & Kanno, Y. (2020). Spawning periodicity and synchrony of bluehead chub (*Nocomis leptocephalus*) and a nest associate, yellowfin shiner (*Notropis lutipinnis*), across streams. Ecology of Freshwater Fish, 29, 299–310. 10.1111/eff.12515

[ece310700-bib-0037] Koenig, W. D. (1999). Spatial autocorrelation of ecological phenomena. Trends in Ecology & Evolution, 14, 22–25. 10.1016/S0169-5347(98)01533-X 10234243

[ece310700-bib-0038] Koenig, W. D. (2001). Synchrony and periodicity of eruptions by boreal birds. The Condor, 103, 725–735. 10.1093/condor/103.4.725

[ece310700-bib-0039] Lahoz‐Monfort, J. J. , Morgan, B. J. T. , Harris, M. P. , Daunt, F. , Wanless, S. , & Freeman, S. N. (2013). Breeding together: Modeling synchrony in productivity in a seabird community. Ecology, 94, 3–10. 10.1890/12-0500.1 23600234

[ece310700-bib-0040] Lahoz‐Monfort, J. J. , Morgan, B. J. T. , Harris, M. P. , Wanless, S. , & Freeman, S. N. (2011). A capture‐recapture model for exploring multi‐species synchrony in survival. Methods in Ecology and Evolution, 2, 116–124. 10.1111/j.2041-210X.2010.00050.x

[ece310700-bib-0041] Liebhold, A. , Koenig, W. D. , & Bjørnstad, O. N. (2004). Spatial synchrony in population dynamics. Annual Review of Ecology, Evolution, and Systematics, 35, 467–490. 10.1146/annurev.ecolsys.34.011802.132516

[ece310700-bib-0042] Little, A. G. , Loughland, I. , & Seebacher, F. (2020). What do warming waters mean for fish physiology and fisheries? Journal of Fish Biology, 97, 328–340. 10.1111/jfb.14402 32441327

[ece310700-bib-0043] Loreau, M. , & de Mazancourt, C. (2008). Species synchrony and its drivers: Neutral and nonneutral community dynamics in fluctuating environments. The American Naturalist, 172, E48–E66. 10.1086/589746 18598188

[ece310700-bib-0044] Lundberg, P. , Ranta, E. , Ripa, J. , & Kaitala, V. (2000). Population variability in space and time. Trends in Ecology & Evolution, 15, 460–464. 10.1016/S0169-5347(00)01981-9 11050349

[ece310700-bib-0045] Lyons, J. , Zorn, T. , Stewart, J. , Seelbach, P. , Wehrly, K. , & Wang, L. (2009). Defining and characterizing coolwater stream assemblages in Michigan and Wisconsin USA. North American Journal of Fisheries Management, 29, 1130–1151. 10.1577/M08-118.1

[ece310700-bib-0046] Lytle, D. A. , & Poff, N. L. (2004). Adaptation to natural flow regimes. Trends in Ecology & Evolution, 19, 94–100. 10.1016/j.tree.2003.10.002 16701235

[ece310700-bib-0047] Matter, S. F. (2001). Synchrony, extinction, and dynamics of spatially segregated, heterogeneous populations. Ecological Modelling, 141, 217–226. 10.1016/S0304-3800(01)00275-7

[ece310700-bib-0048] Matthews, W. J. , & Marsh‐Matthews, E. (2016). Dynamics of an upland stream fish community over 40 years: Trajectories and support for the loose equilibrium concept. Ecology, 97, 706–719. 10.1890/14-2179.1 27197397

[ece310700-bib-0049] Matthews, W. J. , Marsh‐Matthews, E. , Cashner, R. C. , & Gelwick, F. (2013). Disturbance and trajectory of change in a stream fish community over four decades. Oecologia, 173, 955–969. 10.1007/s00442-013-2646-3 23543217

[ece310700-bib-0050] McKenna, J. E., Jr. , Butryn, R. S. , & McDonald, R. P. (2010). Summer stream water temperature models for Great Lakes streams: New York. Transactions of the American Fisheries Society, 139, 1399–1414. 10.1577/T09-153.1

[ece310700-bib-0051] Mims, M. C. , & Olden, J. D. (2012). Life history theory predicts fish assemblage response to hydrologic regimes. Ecology, 93, 35–45. 10.1890/11-0370.1 22486085

[ece310700-bib-0052] Moran, P. A. P. (1953). The statistical analysis of the Canadian lynx cycle. II. Synchronization and meteorology. Australian Journal of Zoology, 1, 291–298. 10.1071/ZO9530291

[ece310700-bib-0053] Mori, A. S. , Furukawa, T. , & Sasaki, T. (2013). Response diversity determines the resilience of ecosystems to environmental change. Biological Reviews, 88, 349–364. 10.1111/brv.12004 23217173

[ece310700-bib-0054] Muths, E. , Chambert, T. , Schmidt, B. R. , Miller, D. A. W. , Hossack, B. R. , Joly, B. , Grolet, O. , Green, D. M. , Pilliod, D. S. , Cheylan, M. , Fisher, R. N. , McCaffery, R. M. , Adams, M. J. , Palen, W. J. , Arntzen, J. W. , Garwood, J. , Fellers, G. , Thirion, J. M. , Besnard, A. , & Campbell Grant, E. H. (2017). Heterogeneous responses of temperate‐zone amphibian populations to climate change complicates conservation planning. Scientific Reports, 7, 1–10. 10.1038/s41598-017-17105-7 29213103 PMC5719039

[ece310700-bib-0055] Myers, B. J. E. , Dolloff, C. A. , Webster, J. R. , Nislow, K. H. , Fair, B. , & Rypel, A. L. (2017). Fish assemblage production estimates in Appalachian streams across a latitudinal and temperature gradient. Ecology of Freshwater Fish, 27, 363–377. 10.1111/eff.12352

[ece310700-bib-0056] Nakagawa, S. , & Schielzeth, H. (2013). A general and simple method for obtaining R2 from generalized linear mixed‐effects models. Methods in Ecology and Evolution, 4, 133–142. 10.1111/j.2041-210x.2012.00261.x

[ece310700-bib-0057] Nelson, J. A. , Rieger, K. J. , Gruber, D. , Cutler, M. , Buckner, B. , & Oufiero, C. E. (2021). Thermal tolerance of cyprinids among an urban‐rural gradient: Plasticity, repeatability and effects of swimming and temperature shock. Journal of Thermal Biology, 100, 103047. 10.1016/j.jtherbio.2021.103047 34503794

[ece310700-bib-0058] Olmos, M. , Payne, M. R. , Nevoux, M. , Prévost, E. , Chaput, G. , Du Pontavice, H. , Guitton, J. , Sheehan, T. , Mills, K. , & Rivot, E. (2020). Spatial synchrony in the response of a long range migratory species (*Salmo salar*) to climate change in the North Atlantic Ocean. Global Change Biology, 26, 1319–1337. 10.1111/gcb.14913 31701595

[ece310700-bib-0059] Palmqvist, E. , & Lundberg, P. (1998). Population extinctions in correlated environments. Oikos, 83, 359–367. 10.2307/3546850

[ece310700-bib-0060] Paradis, E. , Baillie, S. R. , Sutherland, W. J. , & Gregory, R. D. (1999). Dispersal and spatial scale affect synchrony in spatial population dynamics. Ecology Letters, 2, 114–120. 10.1046/j.1461-0248.1999.22060.x

[ece310700-bib-0061] Poff, N. L. , Allan, J. D. , Bain, M. B. , Karr, J. R. , Prestegaard, K. L. , Richter, B. D. , Sparks, R. E. , & Stromberg, J. C. (1997). The natural flow regime: A paradigm for river conservation and restoration. Bioscience, 47, 769–784. 10.2307/1313099

[ece310700-bib-0062] Poff, N. L. , & Ward, J. V. (1989). Implications of streamflow variability and predictability for lotic community structure: A regional analysis of streamflow patterns. Canadian Journal of Fisheries and Aquatic Sciences, 46, 1805–1817. 10.1139/f89-228

[ece310700-bib-0063] Pregler, K. C. , Hanks, R. D. , Childress, E. S. , Hitt, N. P. , Hocking, D. J. , Letcher, B. H. , Wagner, T. , & Kanno, Y. (2019). State‐space analysis of power to detect regional brook trout population trends over time. Canadian Journal of Fisheries and Aquatic Sciences, 76, 2145–2155. 10.1139/cjfas-2018-0241

[ece310700-bib-0064] R Core Team . (2022). R: A language and environment for statistical computing. R Foundation for Statistical Computing. https://www.R‐project.org/

[ece310700-bib-0065] Raimondo, S. , Turcáni, M. , Patoèka, J. , & Liebhold, A. M. (2004). Interspecific synchrony among foliage‐feeding forest Lepidoptera species and the potential role of generalist predators as synchronizing agents. Oikos, 107, 462–470. 10.1111/j.0030-1299.2004.13449.x

[ece310700-bib-0066] Ranta, E. , Kaitala, V. , Lindström, J. , & Lindén, H. (1995). Synchrony in population dynamics. Proceedings of the Royal Society of London, Series B: Biological Sciences, 262, 113–118. 10.1098/rspb.1995.0184

[ece310700-bib-0067] Robertson, G. S. , Bolton, M. , Morrison, P. , & Monaghan, P. (2015). Variation in population synchrony in a multi‐species seabird community: Response to changes in predator abundance. PLoS One, 10(6), 1–15. 10.1371/journal.pone.0131543 PMC4482655 26115174

[ece310700-bib-0068] Rocha, M. R. , Gaedke, U. , & Vasseur, D. A. (2011). Functionally similar species have similar dynamics. Journal of Ecology, 99, 1453–1459. 10.1111/j.1365-2745.2011.01893.x

[ece310700-bib-0069] Rogers, L. A. , & Schindler, D. E. (2008). Asynchrony in population dynamics of sockeye salmon in Southwest Alaska. Oikos, 117, 1578–1586. 10.1111/j.0030-1299.2008.16758.x

[ece310700-bib-0070] Rohde, F. C. , Arndt, R. G. , Foltz, J. W. , & Quattro, J. M. (2009). Freshwater fishes of South Carolina. University of South Carolina Press.

[ece310700-bib-0071] Schaub, M. , & Royle, J. A. (2014). Estimating true instead of apparent survival using spatial Cormack‐jolly‐Seber models. Methods in Ecology and Evolution, 5, 1316–1326. 10.1111/2041-210X.12134

[ece310700-bib-0072] Schindler, D. E. , Armstrong, J. B. , & Reed, T. E. (2015). The portfolio concept in ecology and evolution. Frontiers in Ecology and the Environment, 13, 257–263. 10.1890/140275

[ece310700-bib-0073] Shimadzu, H. , Dornelas, M. , & Magurran, A. E. (2015). Measuring temporal turnover in ecological communities. Methods in Ecology and Evolution, 6, 1384–1394. 10.1111/2041-210X.12438

[ece310700-bib-0074] Siepielski, A. M. , & McPeek, M. A. (2010). On the evidence for species coexistence: A critique of the coexistence program. Ecology, 91, 3153–3164. 10.1890/10-0154.1 21141177

[ece310700-bib-0075] Spiegelhalter, D. J. , Best, N. G. , Carlin, B. P. , & Van Der Linde, A. (2002). Bayesian measures of model complexity and fit. Journal of the Royal Statistical Society, Series B (Statistical Methodology), 64, 583–639. 10.1111/1467-9868.00353

[ece310700-bib-0076] Stenseth, N. C. , Ehrich, D. , Rueness, E. K. , & Jakobsen, K. S. (2004). The effect of climatic forcing on population synchrony and genetic structuring of the Canadian lynx. Proceedings of the National Academy of Sciences of the United States of America, 101, 6056–6061. 15067131 10.1073/pnas.0307123101 PMC395922

[ece310700-bib-0077] Swallow, B. , King, R. , Buckland, S. T. , & Toms, M. P. (2016). Identifying multispecies synchrony in response to environmental covariates. Ecology and Evolution, 6, 8515–8525. 10.1002/ece3.2518 28031803 PMC5167035

[ece310700-bib-0078] Tavecchia, G. , Minguez, E. , De León, A. , Louzao, M. , & Oro, D. (2008). Living close, doing differently: Small‐scale synchrony in demography of two species of seabirds. Ecology, 89, 77–85. 10.1890/06-0326.1 18376549

[ece310700-bib-0079] Tedesco, P. , & Hugueny, B. (2006). Life history strategies affect climate based spatial synchrony in population dynamics of West African freshwater fishes. Oikos, 115, 117–127. 10.1111/j.2006.0030-1299.14847.x

[ece310700-bib-0080] Terui, A. , Kim, S. , Pregler, K. C. , & Kanno, Y. (2021). Non‐random dispersal in sympatric stream fishes: Influences of natural disturbance and body size. Freshwater Biology, 66, 1865–1875. 10.1111/fwb.13796

[ece310700-bib-0081] Tracy, B. H. , Jenkins, R. E. , & Starnes, W. C. (2013). History of fish investigations in the Yadkin‐Pee Dee River drainage of North Carolina and Virginia with an analysis of nonindigenous species and invasion dynamics of three species of suckers (Catostomidae). Journal of the North Carolina Academy of Science, 129, 82–106. 10.7572/2167-5880-129.3.82

[ece310700-bib-0082] Trenham, P. C. , Koenig, W. D. , Mossman, M. J. , Stark, S. L. , & Jagger, L. A. (2003). Regional dynamics of wetland‐breeding frogs and toads: Turnover and synchrony. Ecological Applications, 13, 1522–1532. 10.1890/02-5206

[ece310700-bib-0083] Vannote, R. L. , Minshall, G. W. , Cummins, K. W. , Sedell, J. R. , & Cushing, C. E. (1980). The river continuum concept. Canadian Journal of Fisheries and Aquatic Sciences, 37, 130–137. 10.1139/f80-017

[ece310700-bib-0084] Vasseur, D. A. , & Fox, J. W. (2007). Environmental fluctuations can stabilize food web dynamics by increasing synchrony. Ecology Letters, 10, 1066–1074. 10.1111/j.1461-0248.2007.01099.x 17727665

[ece310700-bib-0085] Vendrametto Granzotti, R. , Agostinho, A. A. , & Bini, L. M. (2021). Drivers and spatial patterns of population synchrony of fish species in a floodplain. Freshwater Biology, 67, 857–872. 10.1111/fwb.13886

[ece310700-bib-0086] Walter, J. A. , Shoemaker, L. G. , Lany, N. K. , Castorani, M. C. N. , Fey, S. B. , Dudney, J. C. , Gherardi, L. , Portales‐Reyes, C. , Rypel, A. L. , Cottingham, K. L. , Suding, K. N. , Reuman, D. C. , & Hallett, L. M. (2021). The spatial synchrony of species richness and its relationship to ecosystem stability. Ecology, 102, e03486. 10.1002/ecy.3486 34289105 PMC9286696

[ece310700-bib-0087] Walther, G. R. (2010). Community and ecosystem responses to recent climate change. Philosophical Transactions of the Royal Society of London. Series B, Biological Sciences, 365, 2019–2024. 10.1098/rstb.2010.0021 20513710 PMC2880129

[ece310700-bib-0088] Winemiller, K. O. (2005). Life history strategies, population regulation, and implications for fisheries management. Canadian Journal of Fisheries and Aquatic Sciences, 62, 872–885. 10.1139/f05-040

