# Supplementary material for: Temperature variation generates interspecific synchrony but spatial asynchrony in survival for freshwater fish communities
Source: Ecol Evol. 2023 Nov 12;13(11):e10700. doi: 10.1002/ece3.10700 (PMC10641305; doi:10.1002/ece3.10700)
Supplement: Supplementary file 1 — Data S1 [file ECE3-13-e10700-s001.docx]

**Supporting Information:**

**Temperature variation generates interspecific synchrony but spatial asynchrony in survival for freshwater fish communities**

Kasey C. Pregler^1,2^*, Xinyi Lu^1^, George Valentine^1^, Seoghyun Kim^1,2^, Yoichiro Kanno^1,2,3^

^1^Department of Fish, Wildlife, and Conservation Biology; Colorado State University; Fort Collins, CO, 80523, USA

^2^Department of Forestry and Environmental Conservation; Clemson University, Clemson, SC, 29634-0317, USA

^3^ Graduate Degree Program in Ecology; Colorado State University, Fort Collins, CO, 80523, USA

*Corresponding author: yoichiro.kanno@colostate.edu

**Supporting Information 1 (S1) Overview:** Supplemental table and figure results. Page 2.

**Supporting Information 2 (S2) Overview:** Model output eqns. 4 & 6. Page 13.

**Supporting Information 3 (S3) Overview:** JAGS code for survival models. Page 27.

**Supporting Information 1 (S1):**

**S1 Overview:** Supplemental tables and results for survival and synchrony analyses.

**Table of Contents:**

Table S1: Prior distributions for parameters used in survival models.

Table S2: Mark-recapture sample size.

Table S3: DIC results for survival models with covariates.

Table S4: Parameter estimates for survival models with covariates.

Figure S1: Pearson’s r correlation coefficients for abiotic environmental covariates.

Figure S2: CJS model recapture probabilities.

Figure S3: Species within Indian Creek survival comparisons.

Figure S4: Species within Todd Creek survival comparisons.

Figure S5: Indian vs Todd Creek survival comparisons.

Table S1. Prior distributions for parameters used in survival models (a.) $logit\left[ \Phi_{i,t} \right]= \mu_{j\left( i \right)}+ \beta_{j\left( i \right),s(i)}x_{t}$ (b.) $logit\left[ \Phi_{i,t} \right]= \mu_{j\left( i \right),s(i)} {+ \varepsilon1}_{t}+ {\varepsilon2}_{j\left( i \right),t}+{\varepsilon3}_{s\left( i \right),t}+{\varepsilon4}_{j\left( i \right),t,s(i)}$and (c.) $logit\left[ \Phi_{i,t} \right]= \mu_{j\left( i \right),s(i)}+\beta x_{t}+ {\varepsilon1}_{t}+ {\varepsilon2}_{j\left( i \right),t}+{\varepsilon3}_{s\left( i \right),t}+{\varepsilon4}_{j\left( i \right),t,s(i)}$

| **Model** | **Parameter** | **Prior distribution** |
| --- | --- | --- |
| (a.) | $\mu_{j(j)}$ | $Normal(\mu,\sigma^{2})$ |
| (a.) | $\beta_{j(i)}$ | $Normal(0,0.37)$ |
| (b.); (c.) | $\mu_{j(j),s\left( i \right)}$ | $Normal(\mu,\sigma^{2})$ |
| (a.); (b.); (c.) | $p_{j(i),t}$ | $Uniform(0,1)$ |
| (b.); (c.) | ${\varepsilon1}_{t}$ | $Normal(0,\tau_{t})$  $\tau_{t}=1/{\sigma^{2}}$  $\sigma\sim Uniform(0,10)$ |
| (b.); (c.) | ${\varepsilon2}_{j\left( i \right),t}$ | $Normal(0,\tau_{j,t})$  $\tau_{j,t}= 1/{\sigma_{j}^{2}}$  $\sigma_{j,t} \sim Uniform(0,10)$ |
| (b.); (c.) | ${\varepsilon3}_{s\left( i \right),t}$ | $Normal(0,\tau_{s,t})$  $\tau_{s,t}=1/{\sigma_{s}^{2}}$  $\sigma_{s} \sim Uniform(0,10)$ |
| (b.); (c.) | ${\varepsilon4}_{j\left( i \right),s\left( i \right),t}$ | $Normal(0,\tau_{j,s,t})$  $\tau_{j,s,t}= 1/{\sigma_{j,s}^{2}}$  $\sigma_{j,s} \sim Uniform(0,10)$ |
| (c.) | $\beta$ | $Normal(0,1)$ |

Table S2: Number of individuals that received a passive integrated transponder tag for each species and stream in the mark-recapture study.

| Species | Indian | Todd |
| --- | --- | --- |
| BHC | 429 | 3608 |
| CRC | 664 | 195 |
| STJ | 244 | 639 |

Table S3. Deviance information criterion (DIC) and differences in DIC (∆DIC, compared to model with lowest DIC) for each environmental covariate included in the Cormack-Jolly Seber models for (a) Indian and (b) Todd creek.

| (a) Indian Creek |  |  |  | (b) Todd Creek |  |  |
| --- | --- | --- | --- | --- | --- | --- |
| Model parameter | DIC | ∆DIC |  | Model parameter | DIC | ∆DIC |
| Mean temperature | 7347 | 0 |  | Mean temperature | 16783 | 0 |
| Mean water level | 7443 | 96 |  | Mean water level | 17046 | 26 |
| Intercept | 7841 | 494 |  | Intercept | 25365 | 8582 |

Table S4. Posterior mean effect sizes and 95% credible intervals in parentheses, for mean temperature and mean water level to survival in Indian and Todd Creeks. Statistically significant effect sizes are in bold (i.e., the 95% credible interval did not overlap 0).

| **Species** | **Mean temperature** | **Mean water level** |
| --- | --- | --- |
| Indian Creek |  |  |
| BHC | **-0.54 (-0.72, -0.34)** | **0.47 (0.22, 0.71)** |
| CRC | **-0.37 (-0.56, -0.17)** | 0.08 (-0.14, 0.31) |
| STJ | **-0.49 (-0.86, -0.11)** | 0.47 (-0.06, 1.00) |
| Todd Creek |  |  |
| BHC | **-0.82 (-0.98, -0.62)** | **0.19 (0.04, 0.37)** |
| CRC | **-0.71 (-1.20, -0.10)** | 0.16 (-0.21, 0.70) |
| STJ | -0.09 (-0.28, 0.09) | 0.18 (-0.02, 0.39) |

Figure S1: Pearson’s r correlation plot for abiotic environmental covariates (mean water level, maximum water level, low water level, mean water temperature, and maximum water temperature) for (A.) Indian Creek and (B.) Todd Creek. Red stars indicate a correlation value is statistically significant (p > 0.05).

1. Covariate correlations in Indian Creek:


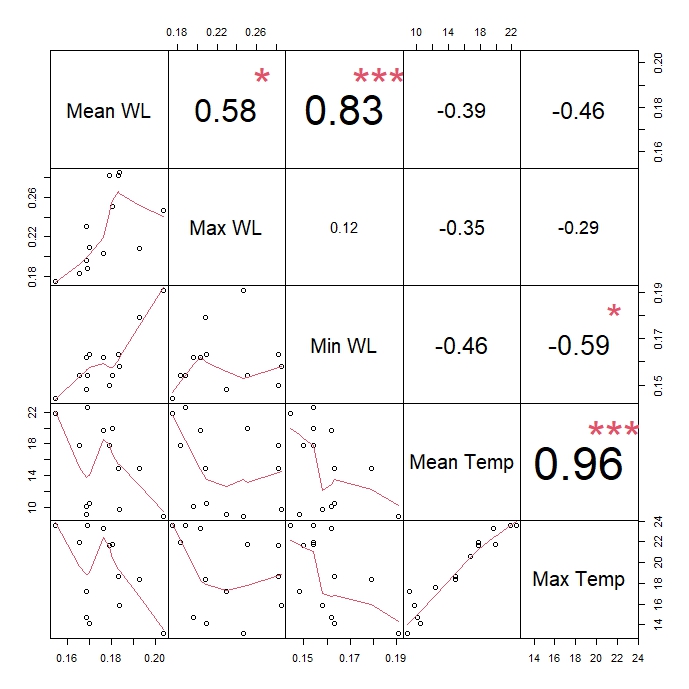


1. Covariate correlations in Todd Creek:


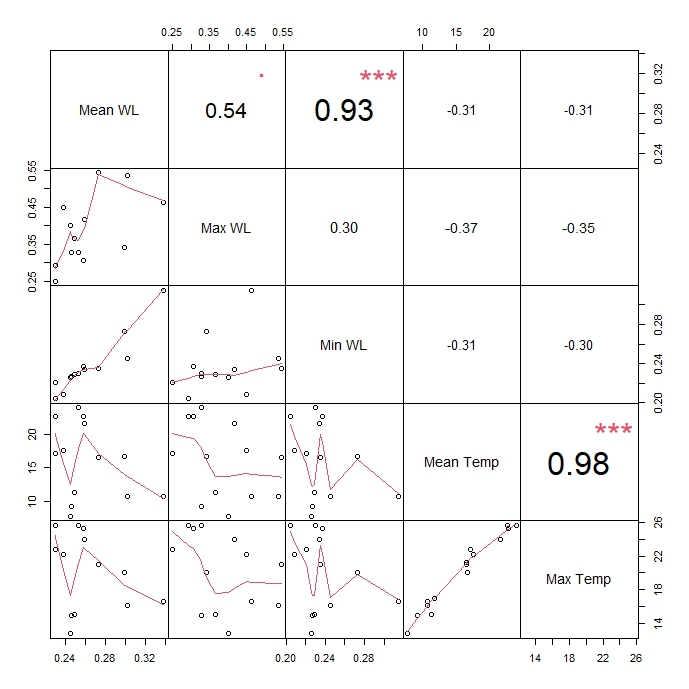


Figure S2: Recapture probabilities for bluehead chub (BHC), creek chub (CRC), striped jumprock (STJ) and occasion from model $\Phi( \varepsilon1+ \varepsilon2+ \varepsilon3+ \varepsilon4$). Note that recapture probabilities in the last occasion (2018-03) is confounded with survival and not identifiable (Lebreton et al. 1992; Kéry & Schwab 2012).


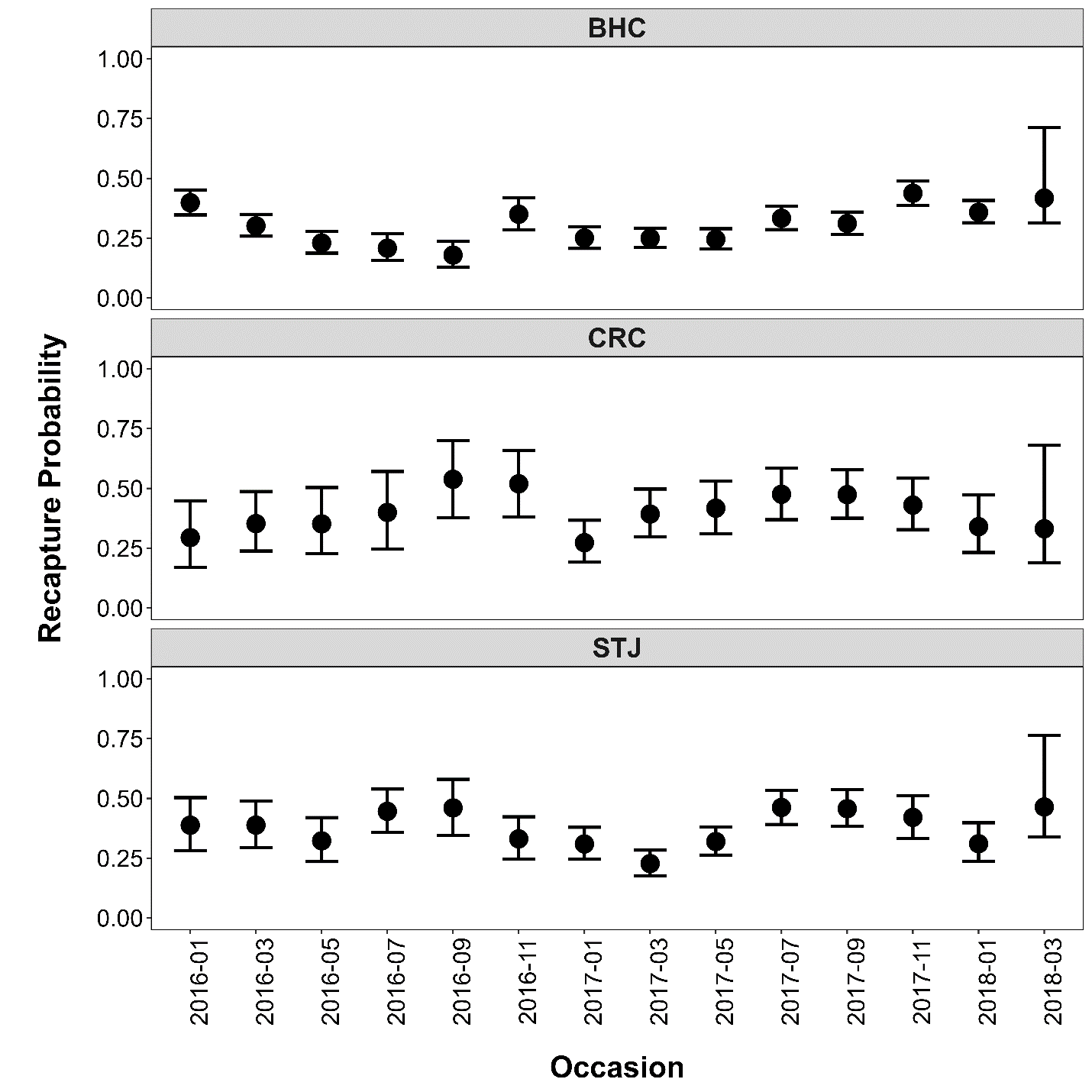


Figure S3: (A.) Among species survival comparisons and (B.) Pearson’s r correlation coefficients within Indian Creek from model $\Phi(\varepsilon1+ \varepsilon2+ \varepsilon3+ \varepsilon4)$. Where “2016-01” represents the first bi-monthly occasion from November 2015 to January 2016. Point estimates are the mean of the MCMC posterior distribution samples for survival of each species in Indian Creek. Vertical bars show 95% credible intervals.


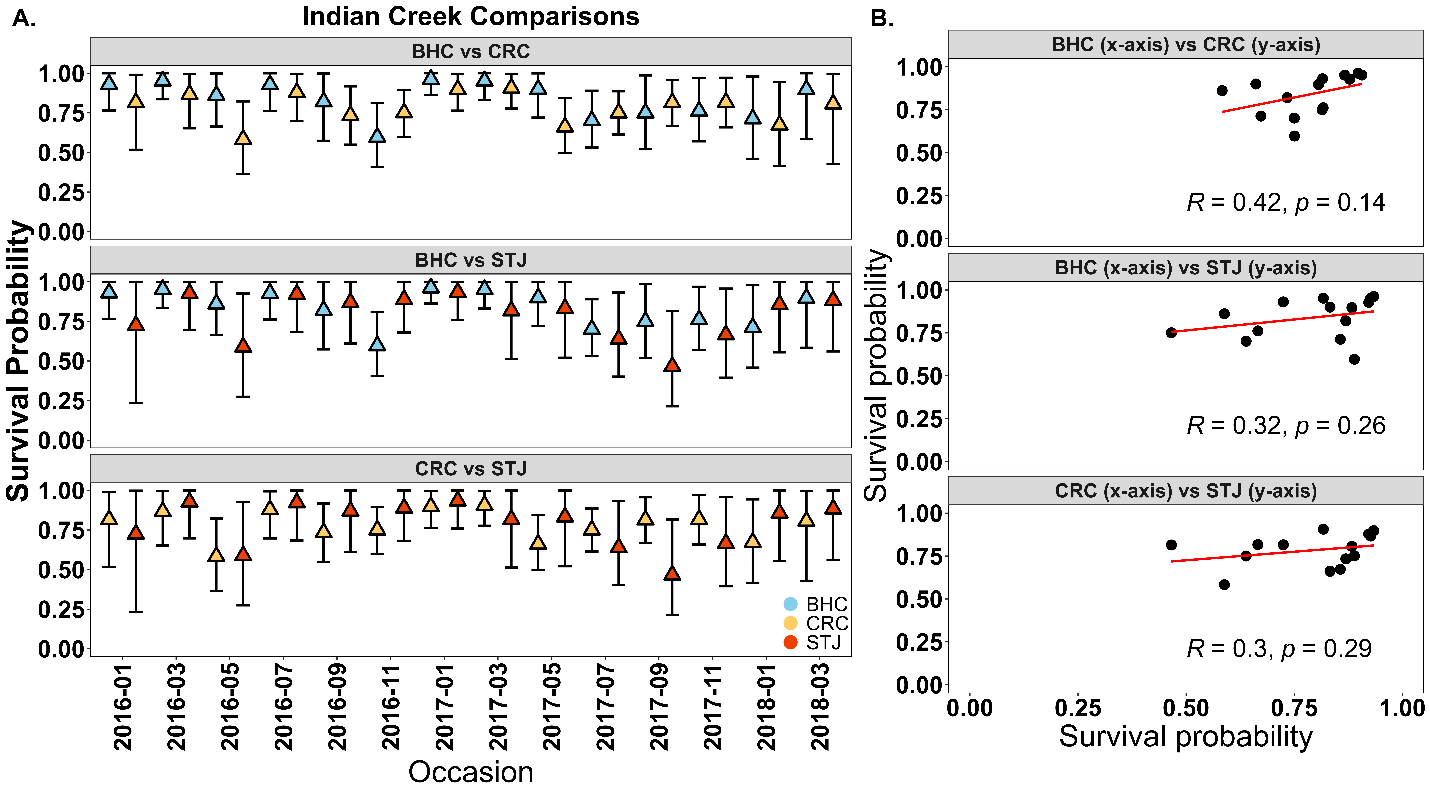


Figure S4: (A.) Among species survival comparisons and (B.) Pearson’s r correlation coefficients within Todd Creek from model $\Phi(\varepsilon1+ \varepsilon2+ \varepsilon3+ \varepsilon4)$. Where “2016-01” represents the first bi-monthly occasion from November 2015 to January 2016. Point estimates are the mean of the MCMC posterior distribution samples for survival of each species in Todd Creek. Vertical bars show 95% credible intervals.


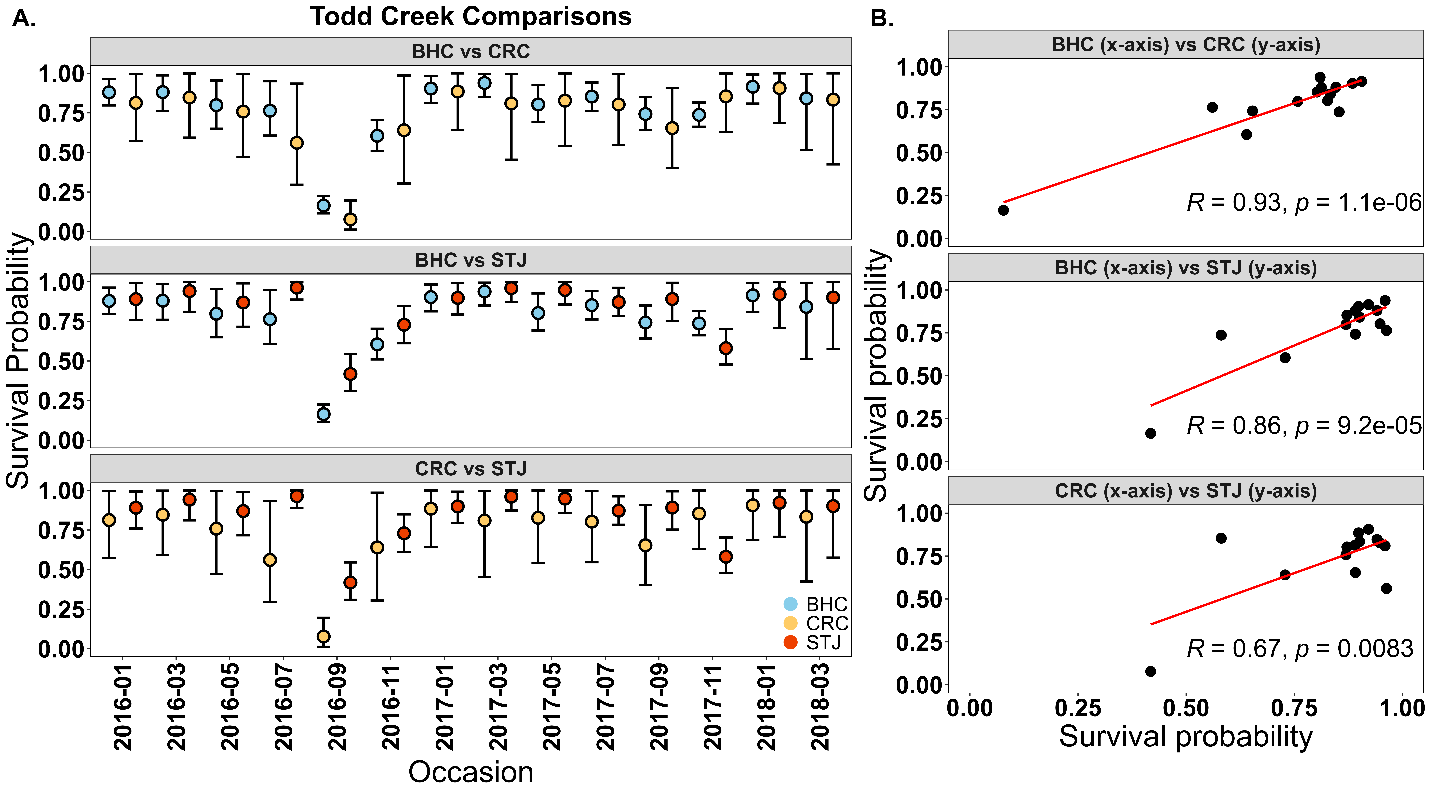


Figure S5: (A.) Among population survival comparisons and (B.) Pearson’s r correlation coefficients for species in Indian vs Todd Creek from model $\Phi(\varepsilon1+ \varepsilon2+ \varepsilon3+ \varepsilon4)$. Where “2016-01” represents the first bi-monthly occasion from November 2015 to January 2016. Point estimates are the mean of the MCMC posterior distribution samples for survival of each species in both streams. Vertical bars show 95% credible intervals.


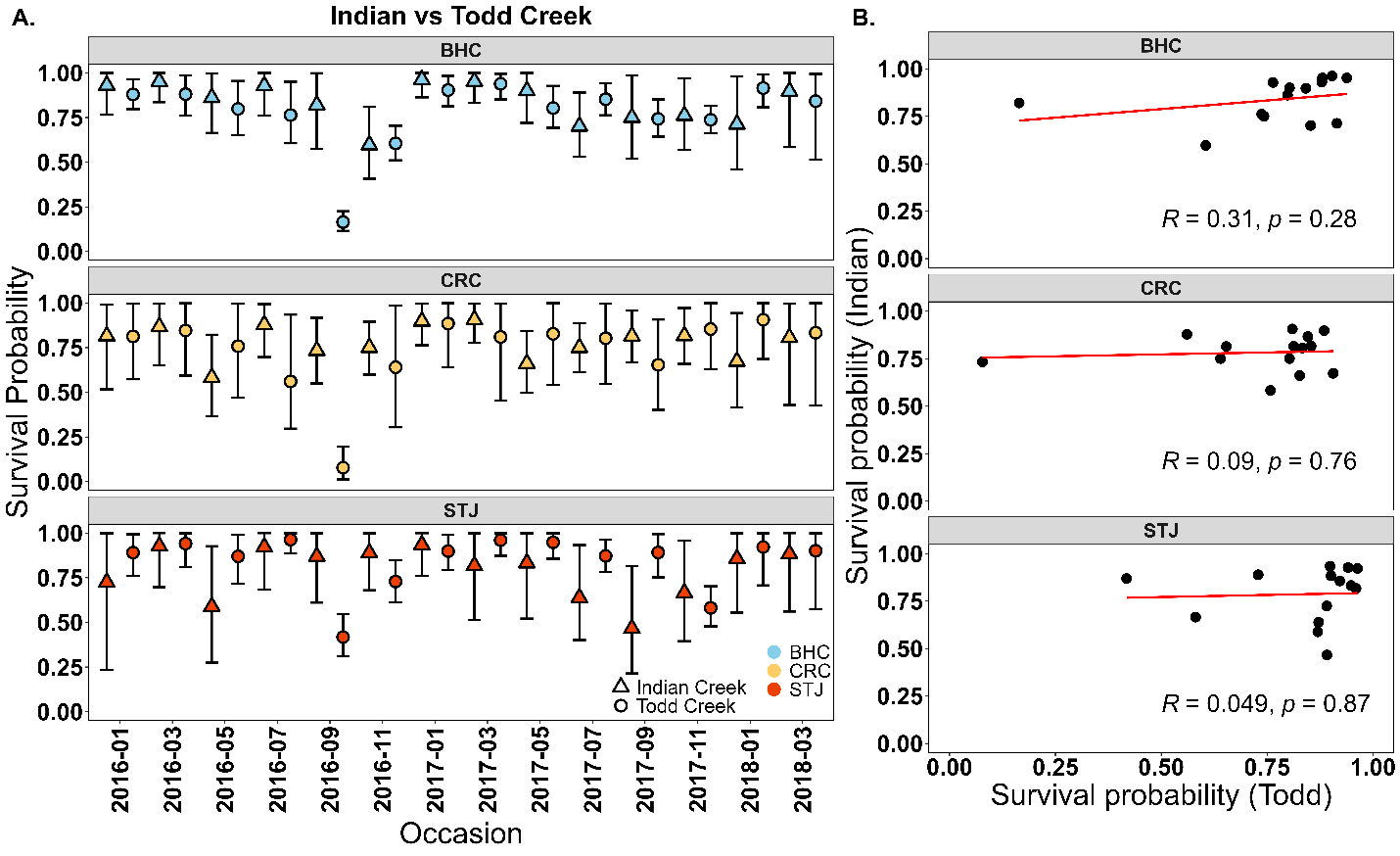


**Supporting information references:**

Kéry M, and M Schaub. 2012. Bayesian population analysis using WinBUGS: a hierarchical perspective. Academic Press*.* Cambridge, Massachusetts.

Lebreton JD, Burnham KP, Clobert J, Anderson DR. 1992. Modeling survival and testing biological hypotheses using marked animals: a unified approach with case studies. Ecological Monographs. 62:67-118. doi: <https://doi.org/10.2307/2937171>

**Supporting Information 2 (S2):**

**S2 Overview:** Model output for spatial survival models (eqns. 4 & 6).

Table S5: Model output for eq. 4

Table S6: Model output for eq. 6

Table S5. Model output for eq. 4: $logit\left[ \Phi_{i,t} \right]= \mu_{j\left( i \right),s\left( i \right)} {+ \varepsilon1}_{t}+ {\varepsilon2}_{j\left( i \right),t}+{\varepsilon3}_{s\left( i \right),t}+{\varepsilon4}_{j\left( i \right),t, s\left( i \right)}$ Model parameters include intercept terms ($\mu$), detection (*p*), random effects ($\varepsilon$1-4), and variance terms (𝜎) and may be indexed by a combination of species (*j*), stream (*s*), and/or time (*t*). Model output includes the posterior mean, and 95% credible interval (2.5-97.5%), standard deviation (SD), potential scale reduction factor ($\hat{R}$), and number of effective samples (n.eff).

| Parameter | Mean | 2.50% | 97.50% | SD | rhat | n.eff |
| --- | --- | --- | --- | --- | --- | --- |
| $\mu_{j,s}$ |  |  |  |  |  |  |
| $\mu$ [BHC,Indian] | 2.404 | 1.222 | 4.979 | 1.057 | 1.019 | 592 |
| $\mu$ [CRC,Indian] | 1.570 | 0.769 | 2.824 | 0.521 | 1.004 | 818 |
| $\mu$ [STJ,Indian] | 2.069 | 0.734 | 4.477 | 0.974 | 1.005 | 4359 |
| $\mu$ [BHC,Todd] | 1.513 | 0.666 | 2.459 | 0.447 | 1.001 | 3491 |
| $\mu$ [CRC,Todd] | 1.504 | 0.245 | 3.742 | 0.970 | 1.007 | 3325 |
| $\mu$ [STJ,Todd] | 2.344 | 1.196 | 3.994 | 0.703 |  | 3791 |
|  |  |  |  |  |  |  |
| *p_j,t_* |  |  |  |  |  |  |
| *p*[BHC, 1] | 0.398 | 0.347 | 0.451 | 0.026 | 1 | 9904 |
| *p*[BHC, 2] | 0.301 | 0.258 | 0.349 | 0.023 | 1 | 92926 |
| *p*[BHC, 3] | 0.230 | 0.187 | 0.278 | 0.023 | 1 | 13268 |
| *p*[BHC, 4] | 0.208 | 0.156 | 0.269 | 0.028 | 1 | 150000 |
| *p*[BHC, 5] | 0.179 | 0.129 | 0.237 | 0.027 | 1 | 41677 |
| *p*[BHC, 6] | 0.350 | 0.285 | 0.418 | 0.034 | 1 | 150000 |
| *p*[BHC, 7] | 0.251 | 0.207 | 0.298 | 0.023 | 1 | 150000 |
| *p*[BHC, 8] | 0.250 | 0.211 | 0.292 | 0.020 | 1 | 150000 |
| *p*[BHC, 9] | 0.245 | 0.204 | 0.289 | 0.021 | 1 | 20892 |
| *p*[BHC, 10] | 0.333 | 0.286 | 0.383 | 0.024 | 1 | 150000 |
| *p*[BHC, 11] | 0.311 | 0.266 | 0.359 | 0.023 | 1 | 60150 |
| *p*[BHC, 12] | 0.438 | 0.387 | 0.489 | 0.026 | 1 | 6230 |
| *p*[BHC, 13] | 0.358 | 0.314 | 0.407 | 0.023 | 1 | 6168 |
| *p*[BHC, 14] | 0.417 | 0.314 | 0.713 | 0.099 | 1 | 9701 |
|  |  |  |  |  |  |  |
| *p*[CRC, 1] | 0.294 | 0.169 | 0.448 | 0.071 | 1 | 50065 |
| *p*[CRC, 2] | 0.352 | 0.238 | 0.487 | 0.063 | 1 | 13362 |
| *p*[CRC, 3] | 0.351 | 0.227 | 0.503 | 0.071 | 1 | 18216 |
| *p*[CRC, 4] | 0.399 | 0.246 | 0.570 | 0.083 | 1 | 52517 |
| *p*[CRC, 5] | 0.538 | 0.377 | 0.700 | 0.083 | 1 | 150000 |
| *p*[CRC, 6] | 0.519 | 0.381 | 0.659 | 0.071 | 1 | 150000 |
| *p*[CRC, 7] | 0.273 | 0.191 | 0.366 | 0.044 | 1 | 71203 |
| *p*[CRC, 8] | 0.392 | 0.297 | 0.497 | 0.051 | 1 | 25238 |
| *p*[CRC, 9] | 0.417 | 0.310 | 0.530 | 0.056 | 1 | 45497 |
| *p*[CRC, 10] | 0.475 | 0.369 | 0.585 | 0.055 | 1 | 35649 |
| *p*[CRC, 11] | 0.473 | 0.375 | 0.577 | 0.510 | 1 | 150000 |
| *p*[CRC, 12] | 0.430 | 0.327 | 0.542 | 0.055 | 1 | 150000 |
| *p*[CRC, 13] | 0.340 | 0.232 | 0.472 | 0.061 | 1 | 26496 |
| *p*[CRC, 14] | 0.331 | 0.189 | 0.680 | 0.119 | 1 | 6553 |
|  |  |  |  |  |  |  |
| *p*[STJ, 1] | 0.387 | 0.282 | 0.503 | 0.056 | 1 | 13117 |
| *p*[STJ, 2] | 0.388 | 0.293 | 0.488 | 0.049 | 1 | 51901 |
| *p*[STJ, 3] | 0.322 | 0.235 | 0.419 | 0.046 | 1 | 108518 |
| *p*[STJ, 4] | 0.445 | 0.357 | 0.538 | 0.046 | 1 | 23108 |
| *p*[STJ, 5] | 0.460 | 0.345 | 0.579 | 0.059 | 1 | 150000 |
| *p*[STJ, 6] | 0.330 | 0.245 | 0.423 | 0.045 | 1 | 150000 |
| *p*[STJ, 7] | 0.309 | 0.245 | 0.379 | 0.034 | 1 | 18478 |
| *p*[STJ, 8] | 0.227 | 0.176 | 0.283 | 0.027 | 1 | 150000 |
| *p*[STJ, 9] | 0.319 | 0.263 | 0.380 | 0.029 | 1 | 7129 |
| *p*[STJ, 10] | 0.462 | 0.391 | 0.534 | 0.036 | 1 | 55310 |
| *p*[STJ, 11] | 0.457 | 0.383 | 0.536 | 0.039 | 1 | 19942 |
| *p*[STJ, 12] | 0.420 | 0.332 | 0.511 | 0.045 | 1 | 128482 |
| *p*[STJ, 13] | 0.310 | 0.237 | 0.398 | 0.040 | 1 | 35954 |
| *p*[STJ, 14] | 0.464 | 0.339 | 0.763 | 0.102 | 1 | 9467 |
|  |  |  |  |  |  |  |
| \| ${\varepsilon1}_{t}$ \| \| --- \| |  |  |  |  |  |  |
| $\varepsilon1$ [1] | 0.125 | -0.899 | 1.354 | 0.532 | 1.001 | 2192 |
| $\varepsilon1$ [2] | 0.336 | -0.621 | 1.854 | 0.615 | 1 | 10706 |
| $\varepsilon1$ [3] | -0.249 | -1.486 | 0.696 | 0.531 | 1 | 17415 |
| $\varepsilon1$ [4] | 0.196 | -0.840 | 1.523 | 0.565 | 1.003 | 624 |
| $\varepsilon1$ [5] | -0.595 | -2.701 | 0.409 | 0.801 | 1.007 | 698 |
| $\varepsilon1$ [6] | -0.302 | -1.620 | 0.573 | 0.547 | 1.001 | 1908 |
| $\varepsilon1$ [7] | 0.385 | -0.529 | 1.786 | 0.584 | 1 | 69775 |
| $\varepsilon1$ [8] | 0.447 | -0.486 | 2.044 | 0.651 | 1 | 28157 |
| $\varepsilon1$ [9] | -0.081 | -1.180 | 0.924 | 0.498 | 1 | 12125 |
| $\varepsilon1$ [10] | -0.140 | -1.248 | 0.823 | 0.492 | 1 | 19464 |
| $\varepsilon1$ [11] | -0.174 | -1.348 | 0.754 | 0.506 | 1 | 5209 |
| $\varepsilon1$ [12] | -0.179 | -1.360 | 0.753 | 0.510 | 1.001 | 2519 |
| $\varepsilon1$ [13] | 0.008 | -1.115 | 1.304 | 0.564 | 1.001 | 2331 |
| $\varepsilon1$ [14] | 0.195 | -1.020 | 1.874 | 0.682 | 1.002 | 2167 |
|  |  |  |  |  |  |  |
| ${\varepsilon2}_{j,t}$ |  |  |  |  |  |  |
| $\varepsilon2$ [BHC,1] | 0.141 | -0.648 | 1.241 | 0.447 | 1 | 150000 |
| $\varepsilon2$ [BHC,2] | 0.093 | -0.805 | 1.230 | 0.473 | 1 | 10350 |
| $\varepsilon2$ [BHC,3] | 0.063 | -0.831 | 1.084 | 0.447 | 1 | 4815 |
| $\varepsilon2$ [BHC,4] | -0.038 | -1.093 | 0.909 | 0.462 | 1 | 2433 |
| $\varepsilon2$ [BHC,5] | -0.215 | -1.606 | 0.578 | 0.519 | 1 | 9103 |
| $\varepsilon2$ [BHC,6] | -0.220 | -1.419 | 0.498 | 0.466 | 1 | 72785 |
| $\varepsilon2$ [BHC,7] | 0.179 | -0.618 | 1.416 | 0.490 | 1 | 26302 |
| $\varepsilon2$ [BHC,8] | 0.225 | -0.574 | 1.606 | 0.530 | 1 | 17435 |
| $\varepsilon2$ [BHC,9] | -0.041 | -1.041 | 0.841 | 0.431 | 1 | 77299 |
| $\varepsilon2$ [BHC,10] | -0.035 | -0.982 | 0.818 | 0.417 | 1 | 80687 |
| $\varepsilon2$ [BHC,11] | -0.137 | -1.222 | 0.636 | 0.437 | 1 | 18553 |
| $\varepsilon2$ [BHC,12] | -0.086 | -1.134 | 0.757 | 0.439 | 1.001 | 6918 |
| $\varepsilon2$ [BHC,13] | 0.002 | -1.003 | 1.019 | 0.467 | 1 | 18154 |
| $\varepsilon2$ [BHC,14] | 0.053 | -0.969 | 1.284 | 0.52 | 1 | 6789 |
|  |  |  |  |  |  |  |
| $\varepsilon2$ [CRC,1] | 0.009 | -1.031 | 1.121 | 0.502 | 1 | 24232 |
| $\varepsilon2$ [CRC,2] | 0.035 | -0.986 | 1.214 | 0.517 | 1 | 150000 |
| $\varepsilon2$ [CRC,3] | -0.188 | -1.409 | 0.644 | 0.493 | 1 | 19256 |
| $\varepsilon2$ [CRC,4] | -0.056 | -1.180 | 0.958 | 0.502 | 1 | 5399 |
| $\varepsilon2$ [CRC,5] | -0.196 | -1.470 | 0.619 | 0.502 | 1 | 26124 |
| $\varepsilon2$ [CRC,6] | 0.003 | -0.897 | 1.013 | 0.443 | 1 | 57443 |
| $\varepsilon2$ [CRC,7] | 0.103 | -0.859 | 1.313 | 0.511 | 1 | 150000 |
| $\varepsilon2$ [CRC,8] | 0.065 | -0.981 | 1.263 | 0.524 | 1 | 22941 |
| $\varepsilon2$ [CRC,9] | -0.153 | -1.358 | 0.683 | 0.488 | 1 | 150000 |
| $\varepsilon2$ [CRC,10] | 0.022 | -0.897 | 0.989 | 0.440 | 1 | 6404 |
| $\varepsilon2$ [CRC,11] | 0.097 | -0.797 | 1.168 | 0.461 | 1 | 13767 |
| $\varepsilon2$ [CRC,12] | 0.260 | -0.519 | 1.660 | 0.545 | 1 | 41685 |
| $\varepsilon2$ [CRC,13] | -0.061 | -1.191 | 0.946 | 0.499 | 1 | 55328 |
| $\varepsilon2$ [CRC,14] | 0.029 | -1.123 | 1.297 | 0.559 | 1 | 28470 |
|  |  |  |  |  |  |  |
| $\varepsilon2$ [STJ,1] | -0.240 | -2.051 | 1.246 | 0.796 | 1 | 26942 |
| $\varepsilon2$ [STJ,2] | 0.290 | -1.183 | 2.490 | 0.901 | 1 | 150000 |
| $\varepsilon2$ [STJ,3] | -0.20 | -1.942 | 1.149 | 0.749 | 1.003 | 1716 |
| $\varepsilon2$ [STJ,4] | 0.672 | -0.665 | 3.177 | 1.008 | 1.001 | 9585 |
| $\varepsilon2$ [STJ,5] | 0.136 | -1.262 | 1.567 | 0.674 | 1.001 | 3895 |
| $\varepsilon2$ [STJ,6] | 0.000009 | -1.441 | 1.409 | 0.674 | 1.001 | 8518 |
| $\varepsilon2$ [STJ,7] | -0.079 | -1.752 | 1.498 | 0.779 | 1.001 | 150000 |
| $\varepsilon2$ [STJ,8] | -0.008 | -1.733 | 1.791 | 0.839 | 1 | 30456 |
| $\varepsilon2$ [STJ,9] | 0.383 | -0.981 | 2.558 | 0.892 | 1.001 | 18563 |
| $\varepsilon2$ [STJ,10] | -0.333 | -2.068 | 0.874 | 0.730 | 1.004 | 2142 |
| $\varepsilon2$ [STJ,11] | -0.220 | -2.002 | 1.224 | 0.773 | 1.003 | 2916 |
| $\varepsilon2$ [STJ,12] | -0.707 | -2.664 | 0.476 | 0.832 | 1 | 11241 |
| $\varepsilon2$ [STJ,13] | 0.166 | -1.482 | 2.354 | 0.924 | 1.001 | 6888 |
| $\varepsilon2$ [STJ,14] | 0.191 | -1.461 | 2.400 | 0.93 | 1.001 | 4996 |
|  |  |  |  |  |  |  |
| ${\varepsilon3}_{s,t}$ |  |  |  |  |  |  |
| $\varepsilon3$[Indian,1] | 0.076 | -1.241 | 1.633 | 0.681 | 1.001 | 21349 |
| $\varepsilon3$[Indian,2] | 0.315 | -0.817 | 2.209 | 0.752 | 1.002 | 3803 |
| $\varepsilon3$[Indian,3] | -0.379 | -2.005 | 0.601 | 0.657 | 1.005 | 775 |
| $\varepsilon3$[Indian,4] | 0.375 | -0.684 | 2.291 | 0.750 | 1.003 | 1740 |
| $\varepsilon3$[Indian,5] | 0.264 | -0.861 | 2.006 | 0.701 | 1.003 | 1934 |
| $\varepsilon3$[Indian,6] | -0.149 | -1.579 | 0.935 | 0.599 | 1.002 | 4329 |
| $\varepsilon3$[Indian,7] | 0.388 | -0.695 | 2.234 | 0.746 | 1.002 | 3232 |
| $\varepsilon3$[Indian,8] | 0.240 | -0.986 | 1.871 | 0.698 | 1.002 | 150000 |
| $\varepsilon3$[Indian,9] | -0.209 | -1.626 | 0.866 | 0.602 | 1.004 | 1296 |
| $\varepsilon3$[Indian,10] | -0.350 | -1.912 | 0.579 | 0.629 | 1.007 | 782 |
| $\varepsilon3$[Indian,11] | -0.217 | -1.711 | 0.832 | 0.613 | 1.004 | 980 |
| $\varepsilon3$[Indian,12] | -0.123 | -1.464 | 0.977 | 0.580 | 1.003 | 2483 |
| $\varepsilon3$[Indian,13] | -0.408 | -2.231 | 0.648 | 0.728 | 1.008 | 709 |
| $\varepsilon3$[Indian,14] | 0.119 | -1.164 | 1.826 | 0.719 | 1.004 | 7094 |
|  |  |  |  |  |  |  |
| $\varepsilon3$[Todd,1] | 0.231 | -1.153 | 1.674 | 0.706 | 1 | 30527 |
| $\varepsilon3$[Todd,2] | 0.254 | -1.249 | 1.974 | 0.799 | 1 | 113162 |
| $\varepsilon3$[Todd,3] | 0.192 | -1.151 | 1.706 | 0.716 | 1.001 | 3334 |
| $\varepsilon3$[Todd,4] | -0.297 | -1.863 | 1.220 | 0.768 | 1.001 | 1318 |
| $\varepsilon3$[Todd,5] | -2.111 | -3.913 | -0.012 | 1.014 | 1.002 | 900 |
| $\varepsilon3$[Todd,6] | -0.541 | -1.918 | 0.747 | 0.677 | 1.001 | 2741 |
| $\varepsilon3$[Todd,7] | 0.225 | -1.240 | 1.799 | 0.754 | 1 | 22917 |
| $\varepsilon3$[Todd,8] | 0.658 | -0.888 | 2.635 | 0.898 | 1 | 38535 |
| $\varepsilon3$[Todd,9] | 0.228 | -1.124 | 1.705 | 0.702 | 1 | 5920 |
| $\varepsilon3$[Todd,10] | 0.365 | -0.882 | 1.740 | 0.656 | 1 | 27091 |
| $\varepsilon3$[Todd,11] | -0.064 | -1.379 | 1.239 | 0.650 | 1 | 19231 |
| $\varepsilon3$[Todd,12] | -0.234 | -1.574 | 1.044 | 0.654 | 1 | 42469 |
| $\varepsilon3$[Todd,13] | 0.925 | -0.543 | 2.993 | 0.912 | 1.001 | 2154 |
| $\varepsilon3$[Todd,14] | 0.320 | -1.232 | 2.452 | 0.911 | 1 | 12084 |
|  |  |  |  |  |  |  |
| ${\varepsilon4}_{j,t,s}$ |  |  |  |  |  |  |
| $\varepsilon4$ [BHC,1, Indian] | 0.581 | -1.581 | 4.139 | 1.503 | 1.002 | 32396 |
| $\varepsilon4$ [BHC,2, Indian] | 0.582 | -1.620 | 4.053 | 1.473 | 1.002 | 13946 |
| $\varepsilon4$ [BHC,3, Indian] | 0.426 | -1.811 | 3.343 | 1.393 | 1.001 | 5542 |
| $\varepsilon4$ [BHC,4, Indian] | 0.336 | -2.160 | 3.719 | 1.481 | 1.001 | 19568 |
| $\varepsilon4$ [BHC,5, Indian] | 0.158 | -2.274 | 3.341 | 1.453 | 1.002 | 29748 |
| $\varepsilon4$ [BHC,6, Indian] | -1.315 | -4.764 | 0.333 | 1.426 | 1.008 | 2402 |
| $\varepsilon4$ [BHC,7, Indian] | 0.686 | -1.412 | 4.325 | 1.497 | 1.001 | 150000 |
| $\varepsilon4$ [BHC,8, Indian] | 0.465 | -1.832 | 3.981 | 1.491 | 1.001 | 150000 |
| $\varepsilon4$ [BHC,9, Indian] | 0.740 | -1.215 | 4.296 | 1.485 | 1.001 | 31704 |
| $\varepsilon4$ [BHC,10, Indian] | -0.970 | -4.179 | 0.615 | 1.323 | 1.009 | 2551 |
| $\varepsilon4$ [BHC,11, Indian] | -0.529 | -3.325 | 1.708 | 1.329 | 1.005 | 10982 |
| $\varepsilon4$ [BHC,12, Indian] | -0.687 | -3.677 | 1.170 | 1.356 | 1.008 | 3192 |
| $\varepsilon4$ [BHC,13, Indian] | -0.888 | -4.219 | 1.075 | 1.431 | 1.008 | 1155 |
| $\varepsilon4$ [BHC,14, Indian] | 0.319 | -2.046 | 3.873 | 1.482 | 1.001 | 28554 |
|  |  |  |  |  |  |  |
| $\varepsilon4$ [CRC,1, Indian] | 0.029 | -1.392 | 1.619 | 0.711 | 1 | 117631 |
| $\varepsilon4$ [CRC,2, Indian] | 0.029 | -1.381 | 1.632 | 0.712 | 1 | 74625 |
| $\varepsilon4$ [CRC,3, Indian] | -0.390 | -2.091 | 0.571 | 0.670 | 1 | 72510 |
| $\varepsilon4$ [CRC,4, Indian] | 0.228 | -0.930 | 2.030 | 0.721 | 1 | 76328 |
| $\varepsilon4$ [CRC,5, Indian] | 0.043 | -1.197 | 1.403 | 0.606 | 1 | 150000 |
| $\varepsilon4$ [CRC,6, Indian] | 0.001 | -1.226 | 1.187 | 0.558 | 1 | 31989 |
| $\varepsilon4$ [CRC,7, Indian] | 0.039 | -1.396 | 1.496 | 0.678 | 1 | 12813 |
| $\varepsilon4$ [CRC,8, Indian] | 0.266 | -0.914 | 2.062 | 0.759 | 1 | 20803 |
| $\varepsilon4$ [CRC,9, Indian] | -0.424 | -2.219 | 0.508 | 0.698 | 1 | 118524 |
| $\varepsilon4$ [CRC,10, Indian] | 0.038 | -1.083 | 1.231 | 0.544 | 1 | 4428 |
| $\varepsilon4$ [CRC,11, Indian] | 0.312 | -0.682 | 1.956 | 0.668 | 1.001 | 68533 |
| $\varepsilon4$ [CRC,12, Indian] | 0.112 | -1.059 | 1.539 | 0.631 | 1.002 | 150000 |
| $\varepsilon4$ [CRC,13, Indian] | -0.270 | -1.946 | 0.856 | 0.690 | 1.001 | 6465 |
| $\varepsilon4$ [CRC,14, Indian] | -0.037 | -1.557 | 1.523 | 0.724 | 1 | 65161 |
|  |  |  |  |  |  |  |
| $\varepsilon4$ [STJ,1, Indian] | -0.497 | -4.020 | 2.796 | 1.658 | 1.002 | 15761 |
| $\varepsilon4$ [STJ,2, Indian] | 0.549 | -2.091 | 4.514 | 1.651 | 1 | 150000 |
| $\varepsilon4$ [STJ,3, Indian] | -0.789 | -3.948 | 1.268 | 1.341 | 1.002 | 34229 |
| $\varepsilon4$ [STJ,4, Indian] | 0.182 | -2.835 | 3.908 | 1.650 | 1 | 24569 |
| $\varepsilon4$ [STJ,5, Indian] | 0.631 | -1.767 | 4.330 | 1.549 | 1 | 61293 |
| $\varepsilon4$ [STJ,6, Indian] | 1.055 | -1.065 | 4.892 | 1.565 | 1 | 35318 |
| $\varepsilon4$ [STJ,7, Indian] | 0.718 | -1.719 | 4.658 | 1.611 | 1 | 43948 |
| $\varepsilon4$ [STJ,8, Indian] | -0.641 | -4.290 | 2.331 | 1.636 | 1.001 | 5746 |
| $\varepsilon4$ [STJ,9, Indian] | 0.143 | -2.692 | 3.811 | 1.586 | 1 | 46060 |
| $\varepsilon4$ [STJ,10, Indian] | -0.572 | -3.298 | 1.448 | 1.195 | 1.004 | 1472 |
| $\varepsilon4$ [STJ,11, Indian] | -1.581 | -4.998 | 0.355 | 1.453 | 1.002 | 3796 |
| $\varepsilon4$ [STJ,12, Indian] | -0.224 | -2.838 | 2.039 | 1.220 | 1.002 | 2516 |
| $\varepsilon4$ [STJ,13, Indian] | 0.668 | -1.751 | 4.504 | 1.569 | 1 | 150000 |
| $\varepsilon4$ [STJ,14, Indian] | 0.413 | -2.197 | 4.241 | 1.595 | 1 | 99007 |
|  |  |  |  |  |  |  |
| $\varepsilon4$ [BHC,1, Todd] | 0.053 | -0.834 | 1.038 | 0.437 | 1 | 75528 |
| $\varepsilon4$ [BHC,2, Todd] | -0.007 | -1.016 | 0.976 | 0.459 | 1 | 60886 |
| $\varepsilon4$ [BHC,3, Todd] | -0.037 | -1.049 | 0.862 | 0.440 | 1 | 7581 |
| $\varepsilon4$ [BHC,4, Todd] | -0.098 | -1.269 | 0.785 | 0.476 | 1.002 | 1484 |
| $\varepsilon4$ [BHC,5, Todd] | -0.230 | -1.674 | 0.561 | 0.537 | 1 | 13411 |
| $\varepsilon4$ [BHC,6, Todd] | -0.015 | -0.913 | 0.861 | 0.406 | 1 | 5657 |
| $\varepsilon4$ [BHC,7, Todd] | 0.076 | -0.802 | 1.152 | 0.454 | 1 | 56663 |
| $\varepsilon4$ [BHC,8, Todd] | 0.152 | -0.696 | 1.451 | 0.509 | 1 | 46979 |
| $\varepsilon4$ [BHC,9, Todd] | -0.156 | -1.340 | 0.619 | 0.465 | 1.001 | 2290 |
| $\varepsilon4$ [BHC,10, Todd] | 0.110 | -0.696 | 1.175 | 0.434 | 1.001 | 4740 |
| $\varepsilon4$ [BHC,11, Todd] | -0.055 | -1.042 | 0.776 | 0.421 | 1 | 15417 |
| $\varepsilon4$ [BHC,12, Todd] | 0.028 | -0.865 | 0.997 | 0.426 | 1 | 12738 |
| $\varepsilon4$ [BHC,13, Todd] | 0.164 | -0.667 | 1.495 | 0.517 | 1.001 | 5939 |
| $\varepsilon4$ [BHC,14, Todd] | 0.018 | -0.986 | 1.116 | 0.483 | 1 | 150000 |
|  |  |  |  |  |  |  |
| $\varepsilon4$ [CRC,1, Todd] | -0.003 | -2.557 | 2.745 | 1.353 | 1.003 | 14703 |
| $\varepsilon4$ [CRC,2, Todd] | 0.107 | -2.438 | 3.189 | 1.395 | 1.002 | 20500 |
| $\varepsilon4$ [CRC,3, Todd] | 0.291 | -1.876 | 3.493 | 1.406 | 1.001 | 17481 |
| $\varepsilon4$ [CRC,4, Todd] | -1.003 | -4.388 | 0.759 | 1.422 | 1.003 | 1869 |
| $\varepsilon4$ [CRC,5, Todd] | -1.286 | -5.027 | 0.383 | 1.501 | 1.002 | 2035 |
| $\varepsilon4$ [CRC,6, Todd] | 0.131 | -2.066 | 2.772 | 1.272 | 1.001 | 108105 |
| $\varepsilon4$ [CRC,7, Todd] | 0.470 | -1.715 | 4.111 | 1.488 | 1.001 | 9080 |
| $\varepsilon4$ [CRC,8, Todd] | -0.729 | -4.488 | 1.637 | 1.587 | 1.002 | 3569 |
| $\varepsilon4$ [CRC,9, Todd] | 0.633 | -1.378 | 4.344 | 1.495 | 1 | 35778 |
| $\varepsilon4$ [CRC,10, Todd] | 0.029 | -2.291 | 2.887 | 1.348 | 1.001 | 144642 |
| $\varepsilon4$ [CRC,11, Todd] | -0.650 | -3.644 | 0.974 | 1.237 | 1.003 | 3202 |
| $\varepsilon4$ [CRC,12, Todd] | 0.952 | -0.829 | 4.585 | 1.482 | 1 | 10740 |
| $\varepsilon4$ [CRC,13, Todd] | 0.607 | -1.485 | 4.307 | 1.514 | 1.001 | 7213 |
| $\varepsilon4$ [CRC,14, Todd] | 0.361 | -1.949 | 3.939 | 1.485 | 1 | 30630 |
|  |  |  |  |  |  |  |
| $\varepsilon4$ [STJ,1, Todd] | -0.094 | -4.020 | 1.834 | 0.933 | 1.001 | 35265 |
| $\varepsilon4$ [STJ,2, Todd] | 0.153 | -2.091 | 2.638 | 1.080 | 1.001 | 9483 |
| $\varepsilon4$ [STJ,3, Todd] | 0.066 | -3.948 | 2.049 | 0.938 | 1.001 | 35795 |
| $\varepsilon4$ [STJ,4, Todd] | 0.865 | -2.835 | 3.827 | 1.193 | 1 | 128189 |
| $\varepsilon4$ [STJ,5, Todd] | -0.110 | -1.767 | 1.32 | 0.823 | 1.002 | 2624 |
| $\varepsilon4$ [STJ,6, Todd] | -0.486 | -1.065 | 0.788 | 0.850 | 1.002 | 3572 |
| $\varepsilon4$ [STJ,7, Todd] | -0.471 | -1.179 | 1.100 | 0.988 | 1.002 | 11091 |
| $\varepsilon4$ [STJ,8, Todd] | 0.302 | -4.290 | 2.864 | 1.071 | 1.001 | 150000 |
| $\varepsilon4$ [STJ,9, Todd] | 0.498 | -2.692 | 3.099 | 1.065 | 1.001 | 82451 |
| $\varepsilon4$ [STJ,10, Todd] | -0.235 | -3.298 | 1.218 | 0.827 | 1.001 | 10962 |
| $\varepsilon4$ [STJ,11, Todd] | 0.521 | -4.998 | 3.110 | 1.040 | 1.001 | 11796 |
| $\varepsilon4$ [STJ,12, Todd] | -0.890 | -2.838 | 0.450 | 0.952 | 1.001 | 5722 |
| $\varepsilon4$ [STJ,13, Todd] | -0.146 | -1.751 | 2.254 | 1.138 | 1 | 113267 |
| $\varepsilon4$ [STJ,14, Todd] | 0.065 | -2.197 | 2.613 | 1.112 | 1.001 | 70749 |
|  |  |  |  |  |  |  |
| 𝜎^2^ | 0.602 | 0.031 | 1.583 | 0.418 | 1 | 8946 |
|  |  |  |  |  |  |  |
| 𝜎^2^_s_ |  |  |  |  |  |  |
| 𝜎^2^[Indian] | 0.642 | 0.025 | 1.914 | 0.517 | 1.007 | 533 |
| 𝜎^2^ [Todd] | 1.081 | 0.194 | 2.099 | 0.461 | 1.001 | 1118 |
|  |  |  |  |  |  |  |
| 𝜎^2^_j_ |  |  |  |  |  |  |
| 𝜎^2^ [BHC] | 0.427 | 0.017 | 1.287 | 0.344 | 1 | 37162 |
| 𝜎^2^ [CRC] | 0.446 | 0.017 | 1.376 | 0.371 | 1 | 56320 |
| 𝜎^2^ [STJ] | 0.802 | 0.036 | 2.276 | 0.606 | 1 | 2307 |
|  |  |  |  |  |  |  |
| 𝜎^2^_j,s_ |  |  |  |  |  |  |
| 𝜎^2^ [BHC,Indian] | 1.333 | 0.073 | 4.474 | 1.164 | 1.005 | 2292 |
| 𝜎^2^ [CRC,Indian] | 0.609 | 0.024 | 1.881 | 0.515 | 1.002 | 91376 |
| 𝜎^2^ [STJ,Indian] | 1.508 | 0.099 | 4.475 | 1.145 | 1.002 | 3871 |
|  |  |  |  |  |  |  |
| 𝜎^2^ [BHC,Indian] | 0.412 | 0.016 | 1.277 | 0.342 | 1.001 | 3689 |
| 𝜎^2^ [CRC,Indian] | 1.287 | 0.049 | 4.397 | 1.170 | 1.002 | 1779 |
| 𝜎^2^ [STJ,Indian] | 0.984 | 0.047 | 2.859 | 0.751 | 1.002 | 3767 |

Table S6. Model output for eq. 6: $logit\left[ \Phi_{i,t} \right]= \mu_{j\left( i \right),s(i)}+{\beta x}_{t}+ {\varepsilon1}_{t}+ {\varepsilon2}_{j\left( i \right),t}+{\varepsilon3}_{s\left( i \right),t}+{\varepsilon4}_{j\left( i \right),t,s\left( i \right).}$Model parameters include intercept terms ($\mu$), temperature effect size ($\beta$), detection (*p*), random effects ($\varepsilon$1-4), and variance terms (𝜎) and may be indexed by a combination of species (*j*), stream (*s*), and/or time (*t*). Model output includes the posterior mean, and 95% credible interval (2.5-97.5%), standard deviation (SD), potential scale reduction factor ($\hat{R}$), and number of effective samples (n.eff).

| Parameter | Mean | 2.50% | 97.50% | SD | $\hat{R}$ | n.eff |
| --- | --- | --- | --- | --- | --- | --- |
| $\mu_{j,s}$ |  |  |  |  |  |  |
| $\mu$ [BHC,Indian] | 2.399 | 1.354 | 4.403 | 0.787 | 1.002 | 3664 |
| $\mu$ [CRC,Indian] | 1.760 | 0.826 | 3.119 | 0.598 | 1.005 | 6130 |
| $\mu$ [STJ,Indian] | 2.114 | 0.833 | 4.313 | 0.877 | 1 | 6895 |
| $\mu$ [BHC,Todd] | 1.551 | 0.874 | 2.275 | 0.350 | 1.001 | 1316 |
| $\mu$ [CRC,Todd] | 1.482 | 0.424 | 3.345 | 0.769 | 1.005 | 959 |
| $\mu$ [STJ,Todd] | 2.416 | 1.349 | 3.879 | 0.633 | 1 | 2527 |
|  |  |  |  |  |  |  |
| $\beta$ | -0.725 | -1.334 | -0.144 | 0.300 | 1 | 20489 |
|  |  |  |  |  |  |  |
| *p_j,t_* |  |  |  |  |  |  |
| *p*[BHC, 1] | 0.398 | 0.348 | 0.451 | 0.026 | 1 | 150000 |
| *p*[BHC, 2] | 0.298 | 0.256 | 0.345 | 0.022 | 1 | 62556 |
| *p*[BHC, 3] | 0.228 | 0.187 | 0.275 | 0.022 | 1 | 23209 |
| *p*[BHC, 4] | 0.215 | 0.164 | 0.274 | 0.028 | 1 | 29257 |
| *p*[BHC, 5] | 0.181 | 0.130 | 0.239 | 0.028 | 1 | 60023 |
| *p*[BHC, 6] | 0.350 | 0.286 | 0.419 | 0.034 | 1 | 71930 |
| *p*[BHC, 7] | 0.249 | 0.206 | 0.296 | 0.023 | 0.999 | 150000 |
| *p*[BHC, 8] | 0.249 | 0.210 | 0.290 | 0.020 | 1 | 22341 |
| *p*[BHC, 9] | 0.244 | 0.203 | 0.287 | 0.021 | 1 | 71812 |
| *p*[BHC, 10] | 0.334 | 0.287 | 0.383 | 0.024 | 0.999 | 150000 |
| *p*[BHC, 11] | 0.314 | 0.269 | 0.361 | 0.023 | 0.999 | 150000 |
| *p*[BHC, 12] | 0.440 | 0.390 | 0.490 | 0.025 | 1 | 130591 |
| *p*[BHC, 13] | 0.353 | 0.310 | 0.400 | 0.022 | 1 | 34143 |
| *p*[BHC, 14] | 0.437 | 0.308 | 0.528 | 0.06 | 1.012 | 906 |
|  |  |  |  |  |  |  |
| *p*[CRC, 1] | 0.292 | 0.169 | 0.444 | 0.070 | 1 | 150000 |
| *p*[CRC, 2] | 0.342 | 0.233 | 0.473 | 0.061 | 1 | 147696 |
| *p*[CRC, 3] | 0.351 | 0.227 | 0.502 | 0.070 | 1 | 13604 |
| *p*[CRC, 4] | 0.404 | 0.253 | 0.574 | 0.082 | 1 | 150000 |
| *p*[CRC, 5] | 0.541 | 0.38 | 0.704 | 0.083 | 1 | 25288 |
| *p*[CRC, 6] | 0.524 | 0.385 | 0.664 | 0.071 | 1 | 81487 |
| *p*[CRC, 7] | 0.269 | 0.188 | 0.361 | 0.044 | 0.999 | 150000 |
| *p*[CRC, 8] | 0.380 | 0.288 | 0.483 | 0.049 | 1 | 150000 |
| *p*[CRC, 9] | 0.416 | 0.309 | 0.530 | 0.056 | 1 | 69113 |
| *p*[CRC, 10] | 0.477 | 0.371 | 0.588 | 0.055 | 0.999 | 150000 |
| *p*[CRC, 11] | 0.475 | 0.375 | 0.579 | 0.052 | 1 | 33009 |
| *p*[CRC, 12] | 0.432 | 0.326 | 0.547 | 0.056 | 1 | 36812 |
| *p*[CRC, 13] | 0.330 | 0.222 | 0.467 | 0.063 | 1 | 20586 |
| *p*[CRC, 14] | 0.289 | 0.174 | 0.516 | 0.090 | 1.001 | 3673 |
|  |  |  |  |  |  |  |
| *p*[STJ, 1] | 0.387 | 0.281 | 0.502 | 0.056 | 1 | 83757 |
| *p*[STJ, 2] | 0.385 | 0.292 | 0.486 | 0.049 | 1 | 51591 |
| *p*[STJ, 3] | 0.320 | 0.234 | 0.418 | 0.047 | 1 | 150000 |
| *p*[STJ, 4] | 0.445 | 0.356 | 0.539 | 0.046 | 1 | 107429 |
| *p*[STJ, 5] | 0.461 | 0.346 | 0.581 | 0.060 | 1 | 84137 |
| *p*[STJ, 6] | 0.332 | 0.246 | 0.424 | 0.045 | 1 | 150000 |
| *p*[STJ, 7] | 0.309 | 0.245 | 0.377 | 0.033 | 1 | 150000 |
| *p*[STJ, 8] | 0.226 | 0.175 | 0.282 | 0.027 | 1 | 150000 |
| *p*[STJ, 9] | 0.317 | 0.261 | 0.378 | 0.029 | 1 | 150000 |
| *p*[STJ, 10] | 0.463 | 0.392 | 0.535 | 0.036 | 1 | 33399 |
| *p*[STJ, 11] | 0.458 | 0.384 | 0.538 | 0.039 | 1 | 9228 |
| *p*[STJ, 12] | 0.422 | 0.335 | 0.513 | 0.045 | 1 | 38261 |
| *p*[STJ, 13] | 0.330 | 0.236 | 0.393 | 0.040 | 0.999 | 150000 |
| *p*[STJ, 14] | 0.289 | 0.333 | 0.641 | 0.079 | 1.001 | 5386 |
|  |  |  |  |  |  |  |
| \| ${\varepsilon1}_{t}$ \| \| --- \| |  |  |  |  |  |  |
| $\varepsilon1$ [1] | -0.015 | -0.911 | 0.862 | 0.407 | 1 | 61980 |
| $\varepsilon1$ [2] | 0.060 | -0.841 | 1.124 | 0.459 | 1 | 25465 |
| $\varepsilon1$ [3] | -0.126 | -1.101 | 0.625 | 0.406 | 1 | 6108 |
| $\varepsilon1$ [4] | 0.253 | -0.450 | 1.452 | 0.473 | 1 | 7596 |
| $\varepsilon1$ [5] | -0.275 | -1.703 | 0.525 | 0.541 | 1 | 12042 |
| $\varepsilon1$ [6] | -0.181 | -1.179 | 0.513 | 0.407 | 1 | 6596 |
| $\varepsilon1$ [7] | 0.073 | -0.792 | 1.081 | 0.434 | 1 | 60904 |
| $\varepsilon1$ [8] | 0.158 | -0.635 | 1.325 | 0.468 | 1 | 61915 |
| $\varepsilon1$ [9] | -0.024 | -0.884 | 0.785 | 0.386 | 1 | 16679 |
| $\varepsilon1$ [10] | 0.126 | -0.647 | 1.177 | 0.430 | 1 | 10978 |
| $\varepsilon1$ [11] | 0.113 | -0.67 | 1.121 | 0.419 | 1 | 17737 |
| $\varepsilon1$ [12] | -0.063 | -0.922 | 0.708 | 0.384 | 1.001 | 6078 |
| $\varepsilon1$ [13] | -0.106 | -1.207 | 0.811 | 0.468 | 1 | 9212 |
| $\varepsilon1$ [14] | 0.081 | -0.895 | 1.323 | 0.511 | 1 | 1764 |
|  |  |  |  |  |  |  |
| ${\varepsilon2}_{j,t}$ |  |  |  |  |  |  |
| $\varepsilon2$ [BHC,1] | 0.068 | -0.636 | 0.953 | 0.369 | 1 | 11614 |
| $\varepsilon2$ [BHC,2] | 0.013 | -0.801 | 0.862 | 0.386 | 1 | 24269 |
| $\varepsilon2$ [BHC,3] | 0.047 | -0.67 | 0.894 | 0.366 | 1.002 | 2731 |
| $\varepsilon2$ [BHC,4] | 0.047 | -0.693 | 0.898 | 0.368 | 1.001 | 4713 |
| $\varepsilon2$ [BHC,5] | -0.143 | -1.197 | 0.519 | 0.405 | 1.001 | 6055 |
| $\varepsilon2$ [BHC,6] | -0.173 | -1.158 | 0.417 | 0.380 | 1 | 9931 |
| $\varepsilon2$ [BHC,7] | 0.076 | -0.649 | 1.015 | 0.392 | 1.001 | 10153 |
| $\varepsilon2$ [BHC,8] | 0.141 | -0.532 | 1.236 | 0.430 | 1.001 | 20586 |
| $\varepsilon2$ [BHC,9] | -0.021 | -0.819 | 0.713 | 0.350 | 1 | 33117 |
| $\varepsilon2$ [BHC,10] | 0.058 | -0.648 | 0.889 | 0.353 | 1 | 14256 |
| $\varepsilon2$ [BHC,11] | -0.005 | -0.784 | 0.744 | 0.350 | 1 | 37451 |
| $\varepsilon2$ [BHC,12] | -0.062 | -0.902 | 0.620 | 0.355 | 1 | 30099 |
| $\varepsilon2$ [BHC,13] | -0.041 | -0.953 | 0.773 | 0.402 | 1 | 18690 |
| $\varepsilon2$ [BHC,14] | 0.028 | -0.835 | 1.002 | 0.430 | 1.003 | 5320 |
|  |  |  |  |  |  |  |
| $\varepsilon2$ [CRC,1] | -0.020 | -1.205 | 1.177 | 0.551 | 1 | 39811 |
| $\varepsilon2$ [CRC,2] | 0.007 | -1.184 | 1.304 | 0.582 | 1 | 47333 |
| $\varepsilon2$ [CRC,3] | -0.193 | -1.515 | 0.743 | 0.54 | 1 | 13141 |
| $\varepsilon2$ [CRC,4] | -0.032 | -1.178 | 1.105 | 0.532 | 1 | 7618 |
| $\varepsilon2$ [CRC,5] | -0.205 | -1.605 | 0.705 | 0.555 | 1 | 23547 |
| $\varepsilon2$ [CRC,6] | 0.041 | -0.954 | 1.121 | 0.486 | 1 | 37290 |
| $\varepsilon2$ [CRC,7] | 0.052 | -1.072 | 1.363 | 0.571 | 1 | 150000 |
| $\varepsilon2$ [CRC,8] | -0.012 | -1.249 | 1.209 | 0.572 | 1 | 150000 |
| $\varepsilon2$ [CRC,9] | -0.144 | -1.406 | 0.822 | 0.532 | 1 | 5608 |
| $\varepsilon2$ [CRC,10] | 0.091 | -0.849 | 1.216 | 0.485 | 1 | 63961 |
| $\varepsilon2$ [CRC,11] | 0.166 | -0.765 | 1.389 | 0.513 | 1 | 17579 |
| $\varepsilon2$ [CRC,12] | 0.307 | -0.54 | 1.827 | 0.594 | 1 | 37603 |
| $\varepsilon2$ [CRC,13] | -0.100 | -1.458 | 1.011 | 0.579 | 1 | 24122 |
| $\varepsilon2$ [CRC,14] | 0.025 | -1.271 | 1.416 | 0.622 | 1 | 150000 |
|  |  |  |  |  |  |  |
| $\varepsilon2$ [STJ,1] | -0.292 | -2.155 | 1.167 | 0.809 | 1 | 9848 |
| $\varepsilon2$ [STJ,2] | 0.214 | -1.303 | 2.351 | 0.886 | 1 | 150000 |
| $\varepsilon2$ [STJ,3] | -0.208 | -1.904 | 1.132 | 0.733 | 1.002 | 4805 |
| $\varepsilon2$ [STJ,4] | 0.732 | -0.570 | 3.267 | 1.013 | 1 | 8232 |
| $\varepsilon2$ [STJ,5] | 0.191 | -1.117 | 1.620 | 0.666 | 1 | 2894 |
| $\varepsilon2$ [STJ,6] | 0.043 | -1.315 | 1.469 | 0.662 | 1 | 2427 |
| $\varepsilon2$ [STJ,7] | -0.165 | -1.882 | 1.368 | 0.780 | 1.001 | 2490 |
| $\varepsilon2$ [STJ,8] | -0.059 | -1.834 | 1.697 | 0.829 | 1 | 25858 |
| $\varepsilon2$ [STJ,9] | 0.356 | -0.998 | 2.48 | 0.865 | 1 | 17758 |
| $\varepsilon2$ [STJ,10] | -0.220 | -1.737 | 0.974 | 0.657 | 1.001 | 3209 |
| $\varepsilon2$ [STJ,11] | -0.117 | -1.702 | 1.327 | 0.715 | 1 | 27594 |
| $\varepsilon2$ [STJ,12] | -0.642 | -2.450 | 0.514 | 0.792 | 1.005 | 911 |
| $\varepsilon2$ [STJ,13] | 0.079 | -1.661 | 2.161 | 0.904 | 1.001 | 21360 |
| $\varepsilon2$ [STJ,14] | 0.132 | -1.597 | 2.305 | 0.928 | 1 | 150000 |
|  |  |  |  |  |  |  |
| ${\varepsilon3}_{s,t}$ |  |  |  |  |  |  |
| $\varepsilon3$[Indian,1] | 0.014 | -1.599 | 1.825 | 0.805 | 1 | 71625 |
| $\varepsilon3$[Indian,2] | 0.288 | -1.124 | 2.408 | 0.855 | 1 | 38082 |
| $\varepsilon3$[Indian,3] | -0.479 | -2.252 | 0.646 | 0.731 | 1 | 30876 |
| $\varepsilon3$[Indian,4] | 0.664 | -0.523 | 2.906 | 0.903 | 1 | 4817 |
| $\varepsilon3$[Indian,5] | 0.607 | -0.544 | 2.600 | 0.816 | 1 | 10078 |
| $\varepsilon3$[Indian,6] | -0.176 | -1.663 | 1.005 | 0.636 | 1 | 150000 |
| $\varepsilon3$[Indian,7] | 0.325 | -1.035 | 2.376 | 0.830 | 1 | 150000 |
| $\varepsilon3$[Indian,8] | 0.152 | -1.332 | 2.022 | 0.796 | 1 | 150000 |
| $\varepsilon3$[Indian,9] | -0.248 | -1.828 | 1.005 | 0.680 | 1 | 11942 |
| $\varepsilon3$[Indian,10] | -0.271 | -1.803 | 0.808 | 0.634 | 1 | 14142 |
| $\varepsilon3$[Indian,11] | -0.091 | -1.542 | 1.194 | 0.647 | 1 | 18579 |
| $\varepsilon3$[Indian,12] | -0.164 | -1.574 | 1.061 | 0.627 | 1 | 13056 |
| $\varepsilon3$[Indian,13] | -0.746 | -3.031 | 0.623 | 0.967 | 1 | 9176 |
| $\varepsilon3$[Indian,14] | 0.115 | -1.463 | 2.180 | 0.867 | 1 | 9258 |
|  |  |  |  |  |  |  |
| $\varepsilon3$[Todd,1] | -0.030 | -1.19 | 1.148 | 0.573 | 1 | 13634 |
| $\varepsilon3$[Todd,2] | -0.050 | -1.371 | 1.376 | 0.675 | 1 | 150000 |
| $\varepsilon3$[Todd,3] | 0.067 | -1.063 | 1.309 | 0.587 | 1 | 5207 |
| $\varepsilon3$[Todd,4] | 0.063 | -1.151 | 1.284 | 0.599 | 1 | 1804 |
| $\varepsilon3$[Todd,5] | -1.425 | -3.062 | 0.045 | 0.842 | 1 | 34764 |
| $\varepsilon3$[Todd,6] | -0.419 | -1.563 | 0.607 | 0.553 | 1 | 4123 |
| $\varepsilon3$[Todd,7] | -0.058 | -1.315 | 1.232 | 0.624 | 1 | 15494 |
| $\varepsilon3$[Todd,8] | 0.337 | -0.906 | 1.952 | 0.709 | 1 | 19438 |
| $\varepsilon3$[Todd,9] | 0.141 | -0.953 | 1.346 | 0.569 | 1 | 8012 |
| $\varepsilon3$[Todd,10] | 0.584 | -0.444 | 1.817 | 0.586 | 1.001 | 1851 |
| $\varepsilon3$[Todd,11] | 0.317 | -0.753 | 1.460 | 0.552 | 1 | 12397 |
| $\varepsilon3$[Todd,12] | -0.067 | -1.169 | 0.969 | 0.529 | 1.002 | 2833 |
| $\varepsilon3$[Todd,13] | 0.401 | -0.872 | 2.182 | 0.762 | 1 | 33752 |
| $\varepsilon3$[Todd,14] | 0.194 | -1.195 | 1.978 | 0.780 | 1 | 3968 |
|  |  |  |  |  |  |  |
| ${\varepsilon4}_{j,t,s}$ |  |  |  |  |  |  |
| $\varepsilon4$ [BHC,1, Indian] | 0.432 | -1.588 | 3.591 | 1.308 | 1 | 110470 |
| $\varepsilon4$ [BHC,2, Indian] | 0.395 | -1.697 | 3.510 | 1.304 | 1.001 | 12760 |
| $\varepsilon4$ [BHC,3, Indian] | 0.394 | -1.588 | 3.099 | 1.179 | 1 | 5820 |
| $\varepsilon4$ [BHC,4, Indian] | 0.360 | -1.765 | 3.275 | 1.242 | 1.003 | 12015 |
| $\varepsilon4$ [BHC,5, Indian] | 0.249 | -1.802 | 3.045 | 1.187 | 1.001 | 24181 |
| $\varepsilon4$ [BHC,6, Indian] | -1.087 | -4.023 | 0.350 | 1.178 | 1.001 | 6251 |
| $\varepsilon4$ [BHC,7, Indian] | 0.482 | -1.488 | 3.724 | 1.328 | 1.001 | 17848 |
| $\varepsilon4$ [BHC,8, Indian] | 0.336 | -1.791 | 3.441 | 1.317 | 1.001 | 114482 |
| $\varepsilon4$ [BHC,9, Indian] | 0.609 | -1.135 | 3.762 | 1.272 | 1 | 26206 |
| $\varepsilon4$ [BHC,10, Indian] | -0.671 | -3.227 | 0.722 | 1.023 | 1.002 | 5846 |
| $\varepsilon4$ [BHC,11, Indian] | -0.285 | -2.563 | 1.583 | 1.040 | 1.001 | 5289 |
| $\varepsilon4$ [BHC,12, Indian] | -0.543 | -3.002 | 1.081 | 1.060 | 1.001 | 9235 |
| $\varepsilon4$ [BHC,13, Indian] | -0.853 | -4.114 | 1.148 | 1.356 | 1.001 | 6829 |
| $\varepsilon4$ [BHC,14, Indian] | 0.221 | -2.030 | 3.367 | 1.327 | 1.001 | 8051 |
|  |  |  |  |  |  |  |
| $\varepsilon4$ [CRC,1, Indian] | 0.017 | -1.902 | 2.237 | 1.017 | 1.003 | 71868 |
| $\varepsilon4$ [CRC,2, Indian] | -0.0004 | -1.933 | 2.264 | 1.008 | 1.001 | 16524 |
| $\varepsilon4$ [CRC,3, Indian] | -0.637 | -2.645 | 0.626 | 0.862 | 1.001 | 4549 |
| $\varepsilon4$ [CRC,4, Indian] | 0.518 | -0.959 | 3.002 | 1.012 | 1.001 | 7230 |
| $\varepsilon4$ [CRC,5, Indian] | 0.239 | -1.228 | 1.982 | 0.779 | 1.001 | 9667 |
| $\varepsilon4$ [CRC,6, Indian] | -0.010 | -1.560 | 1.430 | 0.722 | 1.002 | 11983 |
| $\varepsilon4$ [CRC,7, Indian] | -0.074 | -2.043 | 1.918 | 0.949 | 1.001 | 23200 |
| $\varepsilon4$ [CRC,8, Indian] | 0.310 | -1.295 | 2.666 | 0.987 | 1.001 | 14871 |
| $\varepsilon4$ [CRC,9, Indian] | -0.689 | -2.801 | 0.529 | 0.892 | 1.005 | 2565 |
| $\varepsilon4$ [CRC,10, Indian] | 0.164 | -1.203 | 1.638 | 0.698 | 1.005 | 32744 |
| $\varepsilon4$ [CRC,11, Indian] | 0.608 | -0.649 | 2.619 | 0.865 | 1.001 | 38614 |
| $\varepsilon4$ [CRC,12, Indian] | 0.169 | -1.345 | 2.036 | 0.844 | 1.002 | 7977 |
| $\varepsilon4$ [CRC,13, Indian] | -0.604 | -3.114 | 1.029 | 1.066 | 1.002 | 7949 |
| $\varepsilon4$ [CRC,14, Indian] | -0.060 | -2.164 | 2.164 | 1.080 | 1 | 6882 |
|  |  |  |  |  |  |  |
| $\varepsilon4$ [STJ,1, Indian] | -0.505 | -3.989 | 2.593 | 1.591 | 1 | 120784 |
| $\varepsilon4$ [STJ,2, Indian] | 0.366 | -2.225 | 4.056 | 1.521 | 1 | 150000 |
| $\varepsilon4$ [STJ,3, Indian] | -0.716 | -3.743 | 1.255 | 1.255 | 1 | 12627 |
| $\varepsilon4$ [STJ,4, Indian] | 0.223 | -2.557 | 3.690 | 1.497 | 1 | 18507 |
| $\varepsilon4$ [STJ,5, Indian] | 0.694 | -1.459 | 4.182 | 1.415 | 1 | 45934 |
| $\varepsilon4$ [STJ,6, Indian] | 1.008 | -0.924 | 4.676 | 1.458 | 1 | 150000 |
| $\varepsilon4$ [STJ,7, Indian] | 0.533 | -1.839 | 4.25 | 1.499 | 1 | 47409 |
| $\varepsilon4$ [STJ,8, Indian] | -0.630 | -4.264 | 2.252 | 1.585 | 1 | 14794 |
| $\varepsilon4$ [STJ,9, Indian] | 0.099 | -2.610 | 3.590 | 1.498 | 1.001 | 22307 |
| $\varepsilon4$ [STJ,10, Indian] | -0.401 | -2.841 | 1.478 | 1.073 | 1 | 13891 |
| $\varepsilon4$ [STJ,11, Indian] | -1.234 | -4.415 | 0.500 | 1.301 | 1 | 15911 |
| $\varepsilon4$ [STJ,12, Indian] | -0.150 | -2.540 | 1.957 | 1.095 | 1 | 11565 |
| $\varepsilon4$ [STJ,13, Indian] | 0.498 | -1.911 | 4.151 | 1.493 | 1 | 150000 |
| $\varepsilon4$ [STJ,14, Indian] | 0.293 | -2.337 | 3.892 | 1.504 | 1 | 150000 |
|  |  |  |  |  |  |  |
| $\varepsilon4$ [BHC,1, Todd] | 0.016 | -0.808 | 0.858 | 1.308 | 1 | 27117 |
| $\varepsilon4$ [BHC,2, Todd] | -0.042 | -1.007 | 0.817 | 1.304 | 1 | 14172 |
| $\varepsilon4$ [BHC,3, Todd] | -0.028 | -0.896 | 0.780 | 1.179 | 1 | 8684 |
| $\varepsilon4$ [BHC,4, Todd] | -0.031 | -0.935 | 0.750 | 1.242 | 1.001 | 7713 |
| $\varepsilon4$ [BHC,5, Todd] | -0.213 | -1.466 | 0.477 | 1.187 | 1.003 | 1800 |
| $\varepsilon4$ [BHC,6, Todd] | -0.041 | -0.872 | 0.701 | 1.178 | 1 | 7521 |
| $\varepsilon4$ [BHC,7, Todd] | 0.020 | -0.819 | 0.925 | 1.328 | 1 | 150000 |
| $\varepsilon4$ [BHC,8, Todd] | 0.116 | -0.649 | 1.230 | 1.317 | 1.001 | 7837 |
| $\varepsilon4$ [BHC,9, Todd] | -0.128 | -1.183 | 0.568 | 1.272 | 1 | 6753 |
| $\varepsilon4$ [BHC,10, Todd] | 0.172 | -0.487 | 1.251 | 1.023 | 1.001 | 4885 |
| $\varepsilon4$ [BHC,11, Todd] | 0.030 | -0.760 | 0.861 | 1.040 | 1 | 8789 |
| $\varepsilon4$ [BHC,12, Todd] | 0.014 | -0.770 | 0.847 | 1.060 | 1 | 8355 |
| $\varepsilon4$ [BHC,13, Todd] | 0.084 | -0.716 | 1.164 | 1.356 | 1 | 7048 |
| $\varepsilon4$ [BHC,14, Todd] | 0.014 | -0.906 | 1.008 | 1.327 | 1 | 150000 |
|  |  |  |  |  |  |  |
| $\varepsilon4$ [CRC,1, Todd] | -0.048 | -0.245 | 2.379 | 1.017 | 1.003 | 69162 |
| $\varepsilon4$ [CRC,2, Todd] | 0.058 | -0.216 | 2.766 | 1.008 | 1.001 | 17207 |
| $\varepsilon4$ [CRC,3, Todd] | 0.227 | -1.666 | 2.954 | 0.862 | 1.001 | 150000 |
| $\varepsilon4$ [CRC,4, Todd] | -0.734 | -3.533 | 0.761 | 1.012 | 1.003 | 2933 |
| $\varepsilon4$ [CRC,5, Todd] | -1.076 | -4.404 | 0.382 | 0.779 | 1.004 | 1162 |
| $\varepsilon4$ [CRC,6, Todd] | 0.087 | -1.860 | 2.298 | 0.722 | 1.004 | 16519 |
| $\varepsilon4$ [CRC,7, Todd] | 0.336 | -1.627 | 3.384 | 0.949 | 1.002 | 6396 |
| $\varepsilon4$ [CRC,8, Todd] | -0.570 | -3.877 | 1.538 | 0.987 | 1.002 | 2340 |
| $\varepsilon4$ [CRC,9, Todd] | 0.513 | -1.264 | 3.709 | 0.892 | 1.001 | 7114 |
| $\varepsilon4$ [CRC,10, Todd] | 0.051 | -1.953 | 2.420 | 0.698 | 1.003 | 87462 |
| $\varepsilon4$ [CRC,11, Todd] | -0.435 | -3.016 | 1.045 | 0.865 | 1.003 | 1643 |
| $\varepsilon4$ [CRC,12, Todd] | 0.809 | -0.751 | 3.972 | 0.844 | 1.003 | 3067 |
| $\varepsilon4$ [CRC,13, Todd] | 0.449 | -1.421 | 3.627 | 1.066 | 1.003 | 7597 |
| $\varepsilon4$ [CRC,14, Todd] | 0.278 | -1.803 | 3.335 | 1.080 | 1.001 | 13661 |
|  |  |  |  |  |  |  |
| $\varepsilon4$ [STJ,1, Todd] | -0.156 | -0.215 | 1.851 | 1.591 | 1 | 138533 |
| $\varepsilon4$ [STJ,2, Todd] | 0.127 | -2.001 | 2.738 | 1.521 | 1 | 25864 |
| $\varepsilon4$ [STJ,3, Todd] | 0.068 | -1.809 | 2.128 | 1.255 | 1 | 13634 |
| $\varepsilon4$ [STJ,4, Todd] | 1.047 | -0.608 | 4.084 | 1.497 | 1 | 5017 |
| $\varepsilon4$ [STJ,5, Todd] | -0.149 | -2.091 | 1.271 | 1.415 | 1 | 74744 |
| $\varepsilon4$ [STJ,6, Todd] | -0.557 | -2.610 | 0.746 | 1.458 | 1 | 18037 |
| $\varepsilon4$ [STJ,7, Todd] | -0.592 | -2.956 | 1.122 | 1.499 | 1 | 43182 |
| $\varepsilon4$ [STJ,8, Todd] | 0.300 | -1.617 | 2.965 | 1.585 | 1 | 7909 |
| $\varepsilon4$ [STJ,9, Todd] | 0.562 | -1.121 | 3.309 | 1.498 | 1 | 8135 |
| $\varepsilon4$ [STJ,10, Todd] | -0.196 | -2.022 | 1.304 | 1.073 | 1 | 16839 |
| $\varepsilon4$ [STJ,11, Todd] | 0.621 | -0.906 | 3.247 | 1.301 | 1 | 7181 |
| $\varepsilon4$ [STJ,12, Todd] | -0.992 | -3.100 | 0.424 | 1.095 | 1.001 | 1402 |
| $\varepsilon4$ [STJ,13, Todd] | -0.196 | -2.763 | 2.399 | 1.493 | 1 | 11880 |
| $\varepsilon4$ [STJ,14, Todd] | 0.072 | -2.191 | 2.796 | 1.504 | 1 | 12204 |
|  |  |  |  |  |  |  |
| 𝜎^2^ | 0.419 | 0.017 | 1.038 | 0.317 | 1 | 21770 |
|  |  |  |  |  |  |  |
| 𝜎^2^_s_ |  |  |  |  |  |  |
| 𝜎^2^[Indian] | 0.790 | 0.036 | 2.170 | 0.572 | 1 | 8834 |
| 𝜎^2^ [Todd] | 0.811 | 0.106 | 1.665 | 0.384 | 1 | 5278 |
|  |  |  |  |  |  |  |
| 𝜎^2^_j_ |  |  |  |  |  |  |
| 𝜎^2^ [BHC] | 0.339 | 0.012 | 1.038 | 0.282 | 1.002 | 5437 |
| 𝜎^2^ [CRC] | 0.496 | 0.019 | 1.498 | 0.404 | 1 | 20743 |
| 𝜎^2^ [STJ] | 0.787 | 0.039 | 2.206 | 0.586 | 1.001 | 2731 |
|  |  |  |  |  |  |  |
| 𝜎^2^_j,s_ |  |  |  |  |  |  |
| 𝜎^2^ [BHC,Indian] | 0.149 | 0.052 | 3.720 | 0.984 | 1.002 | 5060 |
| 𝜎^2^ [CRC,Indian] | 0.895 | 0.045 | 2.471 | 0.681 | 1.006 | 3126 |
| 𝜎^2^ [STJ,Indian] | 1.374 | 0.076 | 4.119 | 1.066 | 1 | 11402 |
|  |  |  |  |  |  |  |
| 𝜎^2^ [BHC,Indian] | 0.366 | 0.012 | 1.127 | 0.303 | 1.001 | 1730 |
| 𝜎^2^ [CRC,Indian] | 1.092 | 0.046 | 3.692 | 0.995 | 1.004 | 1285 |
| 𝜎^2^ [STJ,Indian] | 1.075 | 0.066 | 2.927 | 0.750 | 1 | 2659 |

**Supporting Information 3 (S3):**

**S3 Overview:** JAGS code for survival analyses.

**Example model code for Eq 2:** $logit\left[ \Phi_{i,t} \right]= \mu_{j\left( i \right)}+ \beta_{j\left( i \right)}x_{t}$

**Single covariate (mean temperature) Cormack-Jolly-Seber model:**

model {

# Priors and constraints

# for (j in 1:nspecies){

for (i in 1:nind){

for (t in f[i]:(n.occasions-1)){ # minus 1 (intervals)

logit(phi[i,t]) <- mu.phi[species[i]]+ a[species[i]]*mean.temp[t]

phi.cor[i,t] <- phi[i,t]^(60/interval[t]) #correction for varying interval lengths

p[i,t] <- mean.p[species[i],t]

} #t

} #i

for(j in 1:3){

a[j]~dnorm(0,0.37)

}

for (j in 1:3){

mu.phi[j] ~ dnorm(0,0.001)T(-1.5,1.5) # prior for logit of mean survival

for(t in 1:(n.occasions-1)){

mean.p[j,t] ~ dunif(0, 1) # Prior for mean recapture

phi.est[j,t] <- 1 / (1+exp(-mu.phi[j]- a[j]*mean.temp[t])) ### Bimonthly survival

}

}

# Likelihood

for (i in 1:nind){

# Define latent state at first capture

z[i,f[i]] <- 1

for (t in (f[i]+1):h[i]){

# State process

z[i,t] ~ dbern(mu1[i,t])

mu1[i,t] <- phi.cor[i,t-1] * z[i,t-1]

# Observation process

y[i,t] ~ dbern(mu2[i,t])

mu2[i,t] <- p[i,t-1] * z[i,t]

} #t

} #i

}

**Example model code for Eq 4:** $logit\left[ \Phi_{i,t} \right]= \mu_{j\left( i \right),s(i)} {+ \varepsilon1}_{t}+ {\varepsilon2}_{j\left( i \right),t}+{\varepsilon3}_{s\left( i \right),t}+{\varepsilon4}_{j\left( i \right),t,s(i)}$

**Spatial synchrony for both Todd and Indian Creek; CJS model with four random effects to estimate synchrony:**

model {

# Priors and constraints

for (i in 1:nind){

for (t in f[i]:(n.occasions-1)){ # minus 1 (intervals)

logit(phi[i,t]) <- mu.phi[species[i],stream[i]] + epsilon[t] + epsilon2[species[i],t] + epsilon3[stream[i],t] + epsilon4[species[i],t,stream[i]]

phi.cor[i,t] <- phi[i,t]^(60/interval[t,stream[i]]) #correction for varying interval lengths

p[i,t] <- mean.p[species[i],t]

}

}

for(t in 1:(n.occasions-1)){

epsilon[t] ~ dnorm(0,tau)

}

tau <- pow(sigma,-2)

sigma ~dunif(0,10)

for(s in 1:2){

tau.str[s] <- pow(sigma2.str[s],-2)

sigma2.str[s] ~ dunif(0,10)

for(t in 1:(n.occasions-1)){

epsilon3[s,t] ~dnorm(0,tau.str[s])

}

}

for(j in 1:3){

tau.sp[j] <- pow(sigma2.sp[j],-2)

sigma2.sp[j] ~ dunif(0,10)

for(t in 1:(n.occasions-1)){

mean.p[j,t] ~ dunif(0, 1) # Prior for mean recapture

epsilon2[j,t] ~dnorm(0,tau.sp[j])

}

}

for(j in 1:3){

for(s in 1:2){

mu.phi[j,s] ~ dnorm(0,0.001) # prior for logit of mean survival

tau.sp.str[j,s] <- pow(sigma.sp.str[j,s],-2)

sigma.sp.str[j,s] ~dunif(0,10)

for(t in 1:(n.occasions-1)){

epsilon4[j,t,s] ~dnorm(0,tau.sp.str[j,s])

}

}

}

# Likelihood

for (i in 1:nind){

# Define latent state at first capture

z[i,f[i]] <- 1

for (t in (f[i]+1):h[i]){

# State process

z[i,t] ~ dbern(mu1[i,t])

mu1[i,t] <- phi.cor[i,t-1] * z[i,t-1]

# Observation process

y[i,t] ~ dbern(mu2[i,t])

mu2[i,t] <- p[i,t-1] * z[i,t]

} #t

} #i

}

**Example model code for Eq. 6:** $logit\left[ \Phi_{i,t} \right]= \mu_{j\left( i \right),s(i)}+\beta x_{t}+ {\varepsilon1}_{t}+ {\varepsilon2}_{j\left( i \right),t}+{\varepsilon3}_{s\left( i \right),t}+{\varepsilon4}_{j\left( i \right),t,s(i)}$

**Spatial synchrony for both Todd and Indian Creek; CJS model with four random effects & mean temperature covariate to estimate contribution of covariates to synchrony:**

model {

# Priors and constraints

for (i in 1:nind){

for (t in f[i]:(n.occasions-1)){ # minus 1 (intervals)

logit(phi[i,t]) <- mu.phi[species[i],stream[i]] +a*mean.temp[t] + epsilon[t] + epsilon2[species[i],t] + epsilon3[stream[i],t] + epsilon4[species[i],t,stream[i]]

phi.cor[i,t] <- phi[i,t]^(60/interval[t,stream[i]]) #correction for varying interval lengths

p[i,t] <- mean.p[species[i],t]

}

}

for(t in 1:(n.occasions-1)){

epsilon[t] ~ dnorm(0,tau)

}

tau <- pow(sigma,-2)

sigma ~dunif(0,10)

for(s in 1:2){

tau.str[s] <- pow(sigma2.str[s],-2)

sigma2.str[s] ~ dunif(0,10)

for(t in 1:(n.occasions-1)){

epsilon3[s,t] ~dnorm(0,tau.str[s])

}

}

for(j in 1:3){

tau.sp[j] <- pow(sigma2.sp[j],-2)

sigma2.sp[j] ~ dunif(0,10)

for(t in 1:(n.occasions-1)){

mean.p[j,t] ~ dunif(0, 1) # Prior for mean recapture

epsilon2[j,t] ~dnorm(0,tau.sp[j])

}

}

a ~ dnorm(0,1)

for(j in 1:3){

for(s in 1:2){

mu.phi[j,s] ~ dnorm(0,0.001) # prior for logit of mean survival

tau.sp.str[j,s] <- pow(sigma.sp.str[j,s],-2)

sigma.sp.str[j,s] ~dunif(0,10)

for(t in 1:(n.occasions-1)){

epsilon4[j,t,s] ~dnorm(0,tau.sp.str[j,s])

}

}

}

# Likelihood

for (i in 1:nind){

# Define latent state at first capture

z[i,f[i]] <- 1

for (t in (f[i]+1):h[i]){

# State process

z[i,t] ~ dbern(mu1[i,t])

mu1[i,t] <- phi.cor[i,t-1] * z[i,t-1]

# Observation process

y[i,t] ~ dbern(mu2[i,t])

mu2[i,t] <- p[i,t-1] * z[i,t]

} #t

} #i

}
